# Hierarchy-Aware AI Nuclear Phenotyping in Human iPSC-Derived Cardiomyocytes: A Preclinical Framework for Imaging Biomarker Development

**DOI:** 10.3390/life16101627

**Published:** 2026-09-29

**Authors:** Md Abu Sufian, Nadeem Qazi, Prashant Ruchaya, Nabeela Berardinelli, Mustansar Ali Ghazanfar, Dipa Baral

**Affiliations:** 1 Department of Engineering and Computing, School of Architecture, Computing and Engineering, University of East London, London E16 2RD, UK; 2 Department of Bioscience, School of Health, Sport and Bioscience, University of East London, London E15 4LZ, UK; 3 Department of Computer Science and Digital Technologies, School of Architecture, Computing and Engineering, University of East London, London E16 2RD, UK

**Keywords:** imaging biomarkers, nuclear phenotyping, artificial intelligence, DINOv2, vision transformer, chromatin texture, fluorescence microscopy, iPSC-derived cardiomyocytes, preclinical imaging biomarkers, translational imaging

## Abstract

**Background/Objectives:** Nuclear morphology and chromatin organisation provide quantifiable imaging features that may serve as candidate biomarkers of cellular state. However, many cellular imaging studies analyse multiple cells from the same microscopy field without explicitly accounting for their hierarchical dependence, which may lead to optimistic estimates of model performance. This study developed a hierarchy-aware AI framework to investigate DAPI-derived nuclear phenotypes in human iPSC-derived cardiomyocytes (iPSC-CMs). **Methods:** A six-day fluorescence microscopy dataset was processed using marker-controlled watershed segmentation, automated quality control, morphology and masked chromatin-texture analysis. Multiple deep-learning, transfer-learning, multiple-instance learning, and self-supervised approaches were evaluated. The final dataset contained 154 quality-controlled nuclei from 51 source FOVs. The primary prediction task was the binary classification of operationally defined Early (Days 1–3) versus Late (Days 4–6) experimental timepoint groups from DAPI-derived nuclear images. Primary evaluation used nested five-fold FOV-grouped cross-validation with three random seeds, preventing nuclei from the same FOV from appearing in both training and test sets. **Results:** For the primary Early-versus-Late timepoint classification task, the prespecified ViT-B/16 model achieved 52.9% accuracy, an F1-score of 0.571, and a ROC-AUC of 0.586. Among secondary full-time-course models, hybrid morphology-gated attention multiple-instance learning achieved the highest accuracy (58.8%) and F1-score (0.644), while frozen-ViT neural pooling achieved the highest ROC-AUC (0.614). In the secondary Days 1–2 versus Days 5–6 extreme-timepoint analysis, the DINOv2 multi-view model achieved 76.7% accuracy, an F1-score of 0.741, and a ROC-AUC of 0.765. FOV-balanced resampling produced similar ROC-AUC estimates for the primary ViT (0.583) and DINOv2 (0.750) analyses. Nuclear area showed the largest FOV-level effect (d=0.59, p=0.0406, q=0.442), with none of the 12 imaging descriptors significant after Benjamini–Hochberg correction. **Conclusions:** DAPI-derived nuclear phenotypes showed a detectable but heterogeneous time-associated imaging signal, with stronger discrimination between more widely separated timepoints. These findings represent candidate imaging signatures rather than validated diagnostic biomarkers. The framework provides a hierarchy-aware approach for prioritising nuclear imaging features for future biological and translational validation.

## 1. Introduction

Quantitative imaging biomarkers are increasingly being investigated as objective measures of cellular and tissue state that may complement conventional molecular and functional assessments in disease modelling, risk stratification, and diagnostic research. In cardiovascular research, human induced pluripotent stem cell-derived cardiomyocytes (iPSC-CMs) provide a translationally relevant experimental platform in which cellular phenotypes can be studied under controlled conditions before evaluation in patient-derived or clinical samples. Nuclear morphology and chromatin organisation are particularly attractive imaging targets because they can be quantified from routinely acquired fluorescence microscopy and may reflect underlying changes in cellular state. Establishing their potential diagnostic relevance, however, requires rigorous preclinical evaluation to determine whether candidate imaging signatures remain detectable across independent experimental sampling units rather than only across individual segmented cells.

Ageing is a fundamental biological process characterised by progressive molecular, cellular, and structural alterations that ultimately impair tissue function and increase susceptibility to disease [1]. Among the cellular hallmarks of ageing, changes in nuclear architecture have emerged as important indicators of cellular dysfunction and senescence. The nucleus is not merely a container for genetic material but a dynamic regulatory structure whose morphology reflects chromatin organisation, gene regulation, and cellular state. Previous studies have associated age-related alterations in nuclear morphology and chromatin organisation with cellular senescence and biological ageing across multiple experimental systems [2,3].

Ageing is accompanied by substantial remodelling of chromatin organisation and genome architecture, including ageing-associated changes in three-dimensional chromatin organisation and higher-order genome structure [4]. Increasing evidence suggests that chromatin reconfiguration and epigenetic alterations play central roles in ageing-associated transcriptional dysregulation, altered cellular responsiveness, and functional decline [5]. Recent studies have reported large-scale chromatin reorganisation during ageing, including changes in chromatin compartmentalisation, topologically associating domains, and nuclear structure [3,6]. Consequently, quantitative assessment of nuclear morphology and chromatin texture has become an important strategy for understanding ageing-associated cellular phenotypes.

Fluorescence microscopy remains one of the most widely used approaches for studying nuclear organisation. In particular, DAPI (4′,6-diamidino-2-phenylindole) staining enables high-contrast visualisation of DNA-rich regions and chromatin distribution within the nucleus. Variations in DAPI fluorescence patterns can provide quantitative information related to DNA distribution, chromatin organisation, and nuclear architecture, providing valuable information regarding cellular state and genome architecture [2]. However, conventional microscopy analysis frequently relies on manual inspection or a restricted set of handcrafted features, which may be insufficient to capture subtle and heterogeneous nuclear phenotypes. Automated quantitative analysis therefore provides an opportunity to investigate whether DAPI-derived nuclear patterns can serve as reproducible candidate imaging biomarkers. Such candidate imaging biomarkers could ultimately complement molecular or functional measurements in translational disease models, provided that their biological specificity and reproducibility are established through independent validation.

Recent advances in artificial intelligence (AI) have transformed biomedical image analysis by enabling automated extraction of complex visual features directly from imaging data. In particular, Vision Transformers (ViTs), originally introduced by Dosovitskiy et al. [7], have emerged as powerful architectures for medical image analysis owing to their ability to model long-range spatial dependencies through self-attention. Transformer-based models are increasingly applied to medical image classification, segmentation, detection, and reconstruction [8], with architectures such as UNETR demonstrating the effectiveness of transformer representations for medical image segmentation [9]. More broadly, recent studies have supported the application of ViT-based approaches across biomedical imaging tasks, including image classification, segmentation, and disease prediction [10,11,12]. From an imaging-biomarker development perspective, however, predictive performance alone is insufficient to establish a validated biomarker. A candidate computational biomarker should demonstrate reproducibility across independent experimental units, resistance to acquisition-related confounding, biologically plausible associations, and ultimately validation against an independent molecular, pathological, or clinical reference standard. These requirements are especially important for preclinical microscopy studies, where large numbers of segmented cells can originate from relatively few independent biological or imaging units.

Despite these advances, the adoption of AI in biomedical research requires not only accurate prediction but also interpretable decision-making. Explainable AI approaches have therefore become increasingly important for understanding how deep learning models identify biologically meaningful image features and for improving trust in AI-assisted biomedical discoveries [13,14,15]. However, applying deep learning to single-cell microscopy data presents an important methodological challenge. Multiple nuclei extracted from the same microscopy field share acquisition conditions and may therefore exhibit within-FOV dependence. If nuclei from the same field of view (FOV) are included in both training and test sets, shared field-specific information may lead to overly optimistic estimates of model generalisation. Attention maps also need to be interpreted carefully, because visually highlighted regions do not necessarily represent image features that truly influence model predictions [16,17].

A related concern in quantitative microscopy is pseudoreplication, which arises when multiple measurements derived from a shared experimental unit are treated as statistically independent observations. In hierarchically structured biological data, failure to account for dependence among nested measurements can artificially increase the apparent sample size and produce overconfident estimates of statistical evidence or model performance [18,19]. This issue is particularly relevant to single-cell imaging, where many segmented nuclei may be obtained from a comparatively small number of microscopy fields.

The development of quantitative imaging biomarkers also requires more than demonstrating statistical separation between experimental groups. Reproducibility of image acquisition, preprocessing, segmentation, feature calculation, and validation has been recognised as a central requirement for translating quantitative image-derived measurements into reliable biomarkers [20,21]. Standardisation initiatives have consequently emphasised transparent feature definitions, benchmarked image-processing procedures, reproducibility assessment, and validation across independent datasets or experimental settings [20]. Although these principles have been developed predominantly in radiomics and clinical imaging, the underlying requirement is equally relevant to quantitative cellular microscopy: an apparent image-derived phenotype should demonstrate reproducibility across independent sampling units before progression toward biological or translational validation.

Despite increasing use of AI for cellular and biomedical image analysis, an important methodological gap remains in the evaluation of nuclear imaging phenotypes derived from hierarchically structured microscopy data. Multiple segmented nuclei are frequently obtained from the same microscopy field, yet cell-level observations may be analysed as though they were fully independent. In addition, relatively few studies combine leakage-controlled field-level validation, quantitative morphology and chromatin-texture analysis, deep-learning comparison, and explanation-faithfulness assessment within a single preclinical imaging-biomarker framework. Addressing these issues is important before image-derived nuclear phenotypes can be prioritised for subsequent biological and translational validation.

The principal novelty of this study lies in a hierarchy-aware preclinical framework for evaluating candidate nuclear imaging biomarkers while explicitly accounting for the nested structure of microscopy data, in which multiple nuclei originate from shared source FOVs. Rather than treating segmented nuclei as independent experimental observations, source-FOV identity is preserved throughout primary model evaluation, statistical inference, and uncertainty estimation. This design addresses an important translational requirement: a candidate imaging signature intended for future biomarker development should generalise beyond individual cells sampled from the same acquisition field. Within this framework, the primary prediction task was the binary classification of operationally defined Early (Days 1–3) versus Late (Days 4–6) time-in-culture groups from DAPI-derived nuclear images under FOV-grouped evaluation. This task was used to determine whether time-associated nuclear imaging differences remained detectable across held-out source FOVs. A secondary extreme-timepoint analysis (Days 1–2 versus Days 5–6) assessed whether greater temporal separation strengthened discrimination, while FOV-level morphology and chromatin-texture analyses evaluated candidate imaging associations. The framework further distinguishes imaging associations from validated biomarkers and spatial attention visualisation from validated explanation faithfulness. The present study should therefore be interpreted as an early-stage preclinical imaging-biomarker investigation rather than validation of a clinical diagnostic test. Its purpose is to establish a rigorous computational and statistical foundation upon which molecularly validated, disease-specific, and ultimately clinically evaluated nuclear imaging biomarkers could subsequently be developed. The principal methodological and translational contributions are summarised in Table 1.

## 2. Materials and Methods

### 2.1. Dataset Acquisition

DAPI-stained fluorescence microscopy images of human induced pluripotent stem cell-derived ventricular cardiomyocytes (axoCells™, Catalogue No. ax2500/ax2508; Axol Bioscience, London, UK) were experimentally acquired in the University of East London Bioscience Laboratory across six experimental time points (Days 1–6). This study used a novel laboratory-generated time-course fluorescence microscopy dataset acquired within a longitudinal cell-culture experiment under controlled experimental conditions for computational analysis of time-associated nuclear phenotypes. Automated marker-controlled watershed segmentation was applied to identify individual nuclei using the watershed transformation introduced by Vincent and Soille [22]. Segmented regions underwent conservative automated quality control based on boundary integrity, area, solidity, circularity, eccentricity, extent, and segmentation-artefact criteria. Following acquisition-level screening, segmentation, region-level quality control, and final dataset-integrity checks, the locked analytical dataset contained 154 quality-controlled nuclei from 51 source FOVs. The final class distribution comprised 94 Early nuclei from 26 FOVs and 60 Late nuclei from 25 FOVs. The acquisition and quality-control flow is summarised in Figure 1. This grouping followed the experimental time course and was used as an operational timepoint-based classification scheme rather than as a definition of two molecularly distinct biological states. The rationale for the Early-versus-Late task was to test whether measurable time-associated changes in nuclear morphology and chromatin organisation emerged across the six-day observation period and remained detectable across held-out source FOVs. Time-dependent differences are biologically plausible because nuclear architecture and chromatin organisation can change with cellular state; however, a sharp distinction between Days 1–3 and Days 4–6 was not assumed a priori. Accordingly, successful discrimination would indicate the presence of a detectable temporal nuclear imaging signal, whereas limited discrimination would indicate substantial phenotypic overlap between the operational timepoint groups. The cell-culture, microscopy acquisition, and experimental sampling characteristics of the dataset are summarised in Table 2.

QC-passed and QC-failed candidate regions were additionally compared across the recorded morphometric descriptors, and threshold robustness was evaluated using relaxed and more stringent morphometric QC specifications while retaining the non-morphological segmentation and artefact safeguards unchanged.

Quality control pipeline details in Table 3.

### 2.2. Multi-Model Nuclear Phenotyping Framework

Quality-controlled nuclear crops were extracted with a 24-pixel margin around each segmented nucleus and centre-padded to a square canvas to preserve the original nuclear aspect ratio. Each square crop was resized to 224×224 pixels using bilinear interpolation. During deep-learning preprocessing, a bicubic 224×224 resize transform was additionally applied to ensure compatibility with the input requirements of the pretrained image backbones; because the extracted nuclear crops were already 224×224 pixels, this step primarily standardised the model-input pipeline. Grayscale images were subsequently replicated across three channels where required for compatibility with ImageNet-pretrained backbones. Four ImageNet-pretrained deep architectures were initially evaluated: ViT-B/16, ResNet50, EfficientNet-B0, and ConvNeXt-Tiny. For each architecture, all pretrained backbone parameters were initially frozen and only the newly configured binary classification head was trained during the three-epoch warm-up stage. During the subsequent fine-tuning stage, the classification head remained trainable and only the final architecture-specific feature stage was unfrozen; all earlier pretrained layers remained frozen. Specifically, the final transformer encoder block was unfrozen for ViT-B/16, layer4 was unfrozen for ResNet50, and the final two feature modules were unfrozen for EfficientNet-B0 and ConvNeXt-Tiny. The models were evaluated using the same outer FOV folds, grouped inner-validation procedure, class-weighted loss, training seeds, and corresponding preprocessing protocol. A parameter-efficient LoRA variant of ViT-B/16 was additionally evaluated. During the initial three-epoch warm-up, the pretrained ViT backbone and LoRA parameters were frozen and only the classification head was trainable. During LoRA adaptation, the ordinary pretrained ViT weights remained frozen, while the classification head and LoRA parameters attached to the fused query-key-value attention projection of the final four transformer blocks were trainable. LoRA used rank r=4, scaling parameter α=8, no LoRA activation dropout, and a classification-head dropout of 0.20. Additional secondary FOV-level approaches included frozen-ViT feature aggregation with neural mean and mean-standard-deviation pooling, attention-based multiple-instance learning, and a hybrid morphology-aware gated-attention model. These FOV-level methods treated all quality-controlled nuclei from a source microscopy field as a bag and generated one probability per FOV. For the multiple-instance approaches, each source FOV constituted one bag and individual nuclei constituted instances within that bag. The morphology-aware gated-attention model incorporated handcrafted nuclear morphology information into the learned FOV-level aggregation, whereas frozen-ViT neural pooling aggregated pretrained image representations without end-to-end backbone fine-tuning. A separate extreme-timepoint sensitivity analysis used a pretrained DINOv2 ViT-S/14 encoder as a frozen feature extractor; its backbone weights were not fine-tuned. Nucleus-level DINOv2 representations were combined with frozen ViT representations, nuclear morphology, and masked chromatin-texture features. For each source FOV, these representations were aggregated using summary statistics and evaluated using nested combinations of logistic regression, linear support vector machines, radial-basis-function support vector machines, principal-component reduction, and top-ranked candidate ensembling. All feature transformation, dimensionality reduction, classifier selection, and ensemble construction were restricted to the corresponding training data, with outer-test FOVs remaining unseen until final evaluation. This secondary analysis was restricted to the more widely separated Days 1–2 versus Days 5–6 timepoints. Consequently, the reported DINOv2 sensitivity results represent pooled out-of-fold predictions from previously unseen source FOVs rather than performance estimated from training or model-selection data.

Accordingly, ViT-B/16 under the Days 1–3 versus Days 4–6 FOV-grouped protocol was defined as the primary deep-learning model and primary performance evaluation. ResNet50, EfficientNet-B0, ConvNeXt-Tiny, LoRA ViT, frozen-ViT pooling, multiple-instance learning, handcrafted feature classifiers, and the DINOv2 extreme-timepoint analysis were treated as secondary comparative or sensitivity analyses and were not used to replace the primary model according to whichever method achieved the highest observed performance.

The training configuration in Table 4 was defined as a fixed modelling protocol rather than randomly assigned during individual model runs. The principal training settings, including a batch size of 8, three warm-up epochs, a maximum of 40 fine-tuning epochs, early-stopping patience of eight epochs, label smoothing of 0.05, and three random seeds (42, 7, and 21), were fixed consistently across the corresponding evaluation pipeline. Architecture-specific backbone and classification-head learning rates were defined for each pretrained model, as reported in Table 4. Training-dependent model selection was restricted to the grouped inner-validation data within each outer fold, including selection of the retained epoch through early stopping. Outer-test FOVs were not used for training or model-selection decisions.

### 2.3. Morphology and Masked Chromatin-Texture Analysis

Six non-duplicated morphology descriptors were evaluated: nuclear area, circularity, eccentricity, solidity, extent, and aspect ratio. Six chromatin-texture descriptors were computed within the nuclear mask: GLCM contrast, dissimilarity, homogeneity, energy, correlation, and chromatin entropy. Images were quantised to 32 intensity levels, and symmetric GLCMs were calculated using pixel distances of 1, 2, and 4 in four directions. Only pixel pairs for which both pixels were located inside the nuclear mask were included. Nucleus-level Mann-Whitney tests were used for candidate biomarker screening, while primary statistical inference was conducted at the source-FOV level. For primary biomarker inference, each descriptor was aggregated using the median within each of the 51 source FOVs used as the primary statistical units. Two-sided class-label permutation testing was then performed with 20,000 Monte Carlo permutations while preserving the numbers of Early and Late FOVs. Effect sizes were reported using Cohen’s *d*, calculated as Late minus Early. Multiple comparisons across the 12 biomarkers were controlled using the Benjamini–Hochberg false discovery rate procedure.

#### 2.3.1. Attention Rollout and Occlusion-Based Faithfulness

Attention rollout was computed for all 154 out-of-fold nuclei using the seed-42 checkpoint corresponding to each nucleus’s held-out FOV fold. Attention matrices were averaged across heads, augmented with identity connections, row-normalised, and recursively multiplied across transformer layers. The resulting class-token-to-patch map was reshaped to the 14×14 ViT patch grid and restricted to patches containing at least 20% nuclear mask occupancy. Faithfulness was assessed by occluding the 20% highest-attention valid nuclear patches and measuring the reduction in the probability of the model’s originally predicted class. This reduction was compared with the mean reduction obtained from 30 size-matched random patch occlusions per nucleus. Nucleus-level effects were averaged within each of the 51 source FOVs before FOV-level statistical testing. A stratified FOV bootstrap with 5000 resamples and a one-sided Wilcoxon signed-rank test evaluated whether attention-targeted occlusion produced a greater probability reduction than random occlusion.

Table 5 summarises the locked dataset composition. The primary and secondary validation protocols are detailed in Section 2.3.2.

Figure 2 summarises the seven-stage leakage-controlled framework. Figure 3 details the leakage-controlled biomarker extraction workflow.

Figure 4 presents representative normalised DAPI microscopy fields and the corresponding marker-controlled watershed segmentation overlays across the six-day experimental time course. Each representative field is shown with a calibrated 20 μm scale bar.

Table 6 summarises the model-validation and statistical-evaluation protocol.

#### 2.3.2. FOV-Grouped Evaluation and Statistical Analysis

The analytical hierarchy comprised nuclei nested within source microscopy FOVs. Source FOV was therefore used as the grouping and primary evaluation unit for the present dataset. This design addresses within-FOV dependence among segmented nuclei. The experiments comprised three independent culture batches as biological replicates, with multiple randomly sampled FOVs providing within-culture image sampling. Because culture-batch identifiers were not encoded in the analytical dataset used for model development, the computational evaluation remained grouped at the source-FOV level. To assess cross-field generalisation while preventing information leakage from shared acquisition fields, the primary evaluation used nested five-fold cross-validation with the source ND2/FOV as the grouping unit. Nested cross-validation was selected to make efficient use of the limited number of source FOVs while maintaining strict separation between training-dependent model selection and final performance estimation. The outer five-fold partition provided held-out source FOVs for performance evaluation, whereas a separate class-balanced grouped inner-validation split within each outer training fold was used for training-dependent and epoch selection. Five outer folds provided a practical balance between retaining sufficient source FOVs for model training and maintaining held-out FOVs for evaluation in each iteration. Class stratification was performed at the source-FOV level so that both Early and Late FOVs were represented across the outer folds, while all nuclei originating from the same source FOV remained within the same outer partition. This prevented nuclei from a shared microscopy field from appearing across training and test sets. The outer-test FOVs remained untouched during training and model selection. Training was repeated using three random seeds. For nucleus-level classifiers evaluated under the FOV-grouped protocol, out-of-fold probabilities were first averaged across nuclei within each held-out FOV and then across seeds to obtain one FOV-level prediction. For a given source FOV *f* containing $N_{f}$ quality-controlled nuclei, the FOV-level predicted probability for the Late class was calculated as

(1) $${\hat{p}}_{f}^{\left(s\right)}=\frac{1}{N_{f}}\sum_{i=1}^{N_{f}}{\hat{p}}_{fi}^{\left(s\right)},$$

where ${\hat{p}}_{fi}^{\left(s\right)}$ denotes the out-of-fold predicted probability for nucleus *i* within FOV *f* under random seed *s*, and $N_{f}$ denotes the number of quality-controlled nuclei belonging to that FOV. Predictions were subsequently averaged across the three random seeds (S=3) to obtain the final FOV-level probability,

(2) $${\bar{p}}_{f}=\frac{1}{S}\sum_{s=1}^{S}{\hat{p}}_{f}^{\left(s\right)}.$$

The final FOV-level class prediction was obtained using the prespecified decision threshold of 0.5. Thus, each source FOV contributed one prediction to the primary performance evaluation, irrespective of the number of nuclei contained within that FOV. For MIL and neural-pooling models, which directly generated one prediction per FOV, predictions were averaged across seeds. Uncertainty around pooled out-of-fold performance was estimated using 5000 stratified bootstrap resamples of the held-out source-FOV predictions, with Early/Late group representation preserved within each resample. The bootstrap was applied after completion of the nested cross-validation pipeline and therefore resampled the resulting out-of-fold FOV-level predictions rather than repeating image preprocessing, model fitting, inner-validation model selection, and outer cross-validation within each bootstrap replicate. Training variability was addressed separately by repeating model training with three random seeds (42, 7, and 21) and averaging the corresponding out-of-fold FOV-level probabilities before bootstrap estimation. Accordingly, the reported bootstrap intervals quantify uncertainty in performance across the available source FOVs conditional on the fitted cross-validation procedure and should not be interpreted as confidence intervals incorporating full pipeline-retraining variability. ROC-AUC was included as a threshold-independent measure of discriminative ability. Unlike accuracy, precision, recall, and F1-score, which were calculated using the prespecified decision threshold of 0.5, ROC-AUC evaluates the ability of predicted probabilities to rank Late FOVs above Early FOVs across all possible classification thresholds. An ROC-AUC of 0.5 represents chance-level discrimination, whereas a value of 1.0 represents perfect discrimination. ROC-AUC was therefore interpreted together with threshold-dependent classification metrics and the Brier score to provide complementary assessment of discrimination and probabilistic prediction. Biomarkers were aggregated using the median within each FOV and compared using 20,000 Monte Carlo class-label permutations with class counts preserved. Out-of-fold attention maps were generated for all 154 nuclei using fold-matched seed-42 checkpoints. Attention faithfulness was assessed by comparing occlusion of the highest-attention 20% of valid nuclear patches with the mean effect of 30 size-matched random occlusions per nucleus.

### 2.4. Day-Specific Performance and Balanced-FOV Sensitivity Analysis

Unequal representation of experimental days may influence pooled performance estimates when the number of source FOVs differs across the time course. The robustness of the primary evaluation to this sampling imbalance was therefore examined using the held-out source-FOV out-of-fold predictions. Performance was first characterised separately for each experimental day using the proportion of correctly classified source FOVs and the mean predicted probability of the Late class. Day-specific ROC-AUC was not estimated because all observations within an individual day belonged to a single operational time-in-culture group, precluding within-day discrimination between the two classes.

Following the day-specific evaluation, the influence of unequal sampling was examined by equalising FOV representation across experimental days. Balancing was performed at the source-FOV level, consistent with the primary evaluation framework and the non-independence of nuclei originating from the same FOV. For the primary Days 1–3 versus Days 4–6 analysis, six FOVs were sampled without replacement from each experimental day, corresponding to the smallest day-specific FOV count. Each balanced resample consequently comprised 36 FOVs, with 18 from the Early and 18 from the Late time-in-culture group. This procedure was repeated for 20,000 resamples using a fixed random seed of 42, with accuracy, precision, recall, F1-score, ROC-AUC, average precision, and Brier score recalculated for each resample.

To determine whether the stronger performance observed in the extreme-timepoint analysis was similarly sensitive to unequal day representation, the balancing procedure was subsequently applied to the DINOv2 analysis. Six FOVs were sampled from each of Days 1, 2, 5, and 6, resulting in 24 FOVs per balanced resample. For both analyses, balanced performance was summarised by the median and the 2.5th–97.5th percentile range across resamples. These resampling analyses quantify the sensitivity of the existing held-out performance estimates to unequal day-specific FOV representation and do not represent independent model training or model-selection experiments.

## 3. Results

Following acquisition screening, segmentation, and quality control, the locked analytical dataset contained 154 nuclei from 51 source FOVs, comprising 94 Early nuclei from 26 FOVs and 60 Late nuclei from 25 FOVs (Table 7). Figure 5 presents representative quality-controlled nucleus crops from each experimental timepoint.

Comparison of QC-passed and QC-failed candidate regions showed that the retained population had higher median circularity and solidity and lower eccentricity and aspect ratio, consistent with removal of geometrically irregular and segmentation-ambiguous candidates. QC-threshold sensitivity analysis produced aggregate retention rates of 42.5%, 39.2%, and 33.2% under relaxed, primary, and stringent morphometric specifications, respectively, without reversal of the Early/Late group-level retention pattern.

Pooled out-of-fold predictions achieved an accuracy of 52.9%, precision of 51.6%, recall of 64.0%, F1-score of 0.571, and a ROC-AUC of 0.586. The ROC-AUC indicates only modest threshold-independent discrimination between the Early and Late source FOVs, with performance relatively close to chance-level ranking (ROC-AUC = 0.5). As shown in Figure 6, the pooled confusion matrix showed that 11 of 26 Early FOVs and 16 of 25 Late FOVs were correctly classified. The pooled Brier score was 0.256. Together, these results provide a conservative estimate of model performance under strict FOV-level separation and emphasise the difficulty of generalising across independent microscopy fields. The leading FOV-level biomarker associations are summarised in Table 8.

The complete FOV-level results for all 12 candidate descriptors are provided.

### 3.1. FOV-Level Candidate Biomarker Analysis

Nucleus-level candidate biomarker screening suggested differences in GLCM dissimilarity (p=0.00085, d=−0.622) and homogeneity (p=0.00343, d=0.595). However, nuclei originating from the same source FOV are not statistically independent. When independent FOVs were used as the statistical units, these associations were not retained after multiple-comparison correction (GLCM dissimilarity: p=0.1473, q=0.442; GLCM homogeneity: p=0.2138, q=0.479). These nucleus-level findings informed candidate biomarker prioritisation, while the FOV-level analysis formed the basis of primary statistical inference. The FOV-level biomarker analysis showed that all morphology and masked chromatin-texture descriptors remained above the significance threshold after correction for 12 comparisons. Nuclear area produced the largest effect and an unadjusted permutation *p*-value below 0.05, but its adjusted *q*-value was 0.442. Given the 51 source FOVs available for primary statistical inference, the analysis had limited power to detect smaller FOV-level effects after correction for multiple comparisons. Moderate FOV-level effects were also observed for GLCM correlation, contrast, dissimilarity, and homogeneity. Aggregating descriptors at the FOV level reduces within-field pseudoreplication and provides a more defensible estimate of cross-field variability.

Table 9 reports pooled out-of-fold performance using source FOVs as the primary evaluation units. Performance differed across evaluation metrics: ConvNeXt-Tiny produced the highest observed accuracy and F1-score, whereas ViT-B/16 produced the highest observed ROC-AUC and average precision and the lowest Brier score. These metric-specific differences demonstrate that model performance depended on the evaluation criterion, with no single architecture showing consistent superiority across all reported measures. ViT-B/16 remained the primary deep-learning model for the principal FOV-grouped evaluation, while the other architectures provided complementary model comparisons.

These architecture comparisons were secondary analyses and were not used to redefine the primary ViT-B/16 model on the basis of the best observed metric. Taken together, these results do not establish reliable visual discrimination between the broad Early (Days 1–3) and Late (Days 4–6) timepoint groups. Performance remained near chance to modest across the evaluated architectures, indicating substantial overlap in DAPI nuclear appearance between the two operational groups under FOV-grouped evaluation. The primary classification results are therefore interpreted as evidence of a weak and heterogeneous time-associated imaging signal, rather than binary visual separation.

The morphology-only classifier produced the strongest handcrafted-feature performance, although discrimination remained modest. Adding masked chromatin-texture features did not improve pooled FOV-level ROC-AUC (Table 10).

The classification performance of the proposed Vision Transformer under the primary FOV-grouped nested five-fold evaluation is summarised in Table 11.

The pooled FOV-level Brier score was 0.256 (95% CI: 0.229–0.287). The Brier score quantifies the mean squared difference between predicted probabilities and observed binary outcomes, with lower values indicating better probabilistic prediction. However, because the present FOV-level classification task was approximately balanced between Early and Late groups, a non-informative model assigning a probability of 0.5 to each class would yield a Brier score of approximately 0.25. Accordingly, the observed value of 0.256 should not be interpreted as evidence of strong probabilistic performance and is consistent with the modest discrimination observed in the primary analysis. The Brier score was used only as an evaluation metric for held-out predictions; it did not influence model optimisation, early stopping, or model selection, which were based on the training objective and grouped inner-validation loss.

### 3.2. Quantitative Attention-Texture Coupling

The attention-texture analysis did not identify statistically supported systematic coupling between ViT attention and local GLCM dissimilarity. The FOV-level observed-minus-null comparison was non-significant (p=0.5486), and the Early-versus-Late comparison was also non-significant (permutation p=0.1178, BH-adjusted q=0.2357; Table 12).

After aggregation to the source-FOV level, the mean observed FOV-median correlation was −0.0098, compared with 0.0008 under the shuffled-texture null. The observed-minus-null difference was not significant (95% bootstrap CI: −0.0810 to 0.0654; Wilcoxon p=0.5486). The Early and Late FOV groups had mean FOV-median correlations of −0.0670 and 0.0497, respectively, corresponding to a Late-minus-Early difference of 0.1166 and Cohen’s d=0.446. This represents a small-to-moderate standardized effect and reflects substantial overlap and heterogeneity in attention-texture coupling across source FOVs rather than a strong separation between the timepoint groups. The group difference was not statistically significant (permutation p=0.1178) and remained non-significant after Benjamini–Hochberg correction (q=0.2357). Therefore, systematic timepoint-dependent coupling between ViT attention and local GLCM dissimilarity was not statistically supported in the locked dataset. The primary FOV-grouped and secondary nucleus-level evaluations are compared in Table 13.

The secondary nucleus-level evaluation produced numerically higher accuracy and ROC-AUC than the primary FOV-grouped analysis (Table 13). These results represent different validation protocols and are presented to illustrate the effect of preserving source-FOV grouping rather than as a formal statistical comparison between the two performance estimates.

The corresponding secondary nucleus-level ViT evaluation is shown in Figure 7.

As shown in Table 14, among the extended full-time-course models, hybrid morphology-gated attention MIL achieved the highest accuracy (0.588), recall (0.760), and F1-score (0.644), whereas frozen-ViT neural pooling achieved the highest ROC-AUC (0.614) and average precision (0.586). Nevertheless, discrimination remained modest across all full-time-course models. The Brier scores provided complementary information on probabilistic prediction. Although the hybrid morphology-gated attention MIL model improved threshold-dependent performance, its Brier score increased to 0.296 compared with 0.256 for the ViT-B/16 model. Because lower Brier scores indicate better agreement between predicted probabilities and observed outcomes, this result shows that the improvement in accuracy, recall, and F1-score did not correspond to improved probability estimates. Similarly, frozen-ViT neural pooling achieved the highest ROC-AUC among the full-time-course models but had a Brier score of 0.273. Thus, improvements in discrimination or threshold-based classification were not consistently accompanied by improved probabilistic performance.

### 3.3. DINOv2 Extreme-Timepoint Sensitivity Analysis

The DINOv2 multi-view ensemble was evaluated as a secondary sensitivity analysis using clearly separated Days 1–2 and Days 5–6. The analysis included 87 nuclei from 30 source FOVs, comprising 17 Early and 13 Late FOVs.

As shown in Table 15, the DINOv2 multi-view ensemble achieved an accuracy of 0.767, an F1-score of 0.741, and a ROC-AUC of 0.765. Fold accuracies ranged from 66.7% to 87.5%. Because this analysis included only 30 source FOVs and deliberately excluded the intermediate Days 3–4, the higher discrimination may partly reflect the easier separation between more temporally distant groups. Accordingly, it was interpreted strictly as a secondary extreme-timepoint sensitivity analysis and not as evidence of strong full-time-course model performance or generalisable biological discrimination.

### 3.4. Day-Specific Performance and Sensitivity to Unequal Day Representation

Day-specific performance of the primary ViT-B/16 model varied across the six experimental days. FOV-level accuracy was 0.286 for Day 1, 0.400 for Day 2, 0.556 for Day 3, 0.667 for Day 4, 0.500 for Day 5, and 0.714 for Day 6. The corresponding numbers of correctly classified FOVs were 2/7, 4/10, 5/9, 8/12, 3/6, and 5/7, respectively. Mean predicted Late-class probabilities were 0.559, 0.502, 0.519, 0.510, 0.538, and 0.565 across Days 1–6, respectively. These day-specific estimates were based on small numbers of FOVs and therefore showed substantial sampling uncertainty.

Sensitivity of FOV-level performance to equal day representation is summarised in Table 16. Balancing the contribution of each experimental day produced performance estimates closely aligned with the original pooled primary analysis. Across 20,000 balanced resamples containing six FOVs per day, the median accuracy was 0.528 (2.5th–97.5th percentile: 0.444–0.611), compared with 0.529 in the original 51-FOV analysis. Median F1-score was 0.564 (0.488–0.650) versus 0.571 originally, and median ROC-AUC was 0.583 (0.494–0.679) versus 0.586 originally. The median Brier score was 0.256, identical after rounding to the original value of 0.256. Thus, equalising the contribution of experimental days did not materially alter the near-chance-to-modest discrimination observed in the primary analysis.

The DINOv2 extreme-timepoint analysis showed a similar pattern. After balancing Days 1, 2, 5, and 6 to six FOVs per day, median accuracy was 0.750 (0.708–0.833), compared with 0.767 in the original 30-FOV analysis, while median ROC-AUC was 0.750 (0.701–0.833), compared with 0.765 originally. Median F1-score was 0.750 (0.720–0.833), compared with 0.741, and median average precision was 0.774 (0.720–0.867), compared with 0.753. These results indicate that the stronger discrimination observed in the extreme-timepoint sensitivity analysis was not explained solely by the unequal number of contributing FOVs across days, although the analysis remains secondary and is based on a small number of source FOVs.

## 4. Discussion

The primary FOV-grouped results indicate that DAPI-derived nuclear appearance alone did not provide reliable binary visual discrimination between the broad Early (Days 1–3) and Late (Days 4–6) timepoint groups. Performance remained near chance to modest across the evaluated architectures, consistent with substantial phenotypic overlap between these adjacent experimental periods. Accordingly, the primary results are more appropriately interpreted as evidence of a weak and heterogeneous time-associated nuclear imaging signal rather than two clearly separable visual phenotypes. In contrast, the secondary DINOv2 extreme-timepoint analysis showed stronger discrimination when the comparison was restricted to Days 1–2 versus Days 5–6. This pattern suggests that time-associated differences in nuclear appearance may become more detectable with wider temporal separation, while intermediate Days 3–4 likely contribute to the overlap observed in the primary Early-versus-Late task. The extreme-timepoint finding therefore supports temporal-separation sensitivity rather than redefining the performance of the primary full-time-course analysis. An important methodological aspect of this study was the use of source FOV as the primary validation unit. Multiple nuclei originated from the same microscopy field and therefore shared acquisition conditions. Treating these nuclei as fully independent validation units could retain field-specific dependence within the evaluation process. The primary analysis therefore preserved source-FOV identity during cross-validation, allowing model performance to be assessed on previously unseen microscopy fields. This effect was evident in the ViT results. The secondary nucleus-level analysis achieved a ROC-AUC of 0.623, which decreased to 0.586 when source-FOV separation was enforced. This reduction should not simply be viewed as poorer model performance. Instead, it provides a more conservative estimate of cross-field generalisation and shows why the underlying sampling hierarchy should be considered when evaluating AI-derived cellular imaging biomarkers.

Attention rollout analysis is illustrated in Figure 8. The attention-texture coupling and occlusion analyses did not provide statistically significant support that the highlighted attention regions represented a specific or faithfully predictive chromatin-texture pattern. Attention rollout was therefore retained only as a spatial visualisation of model attention and should not be interpreted as a faithful or biologically validated explanation of model decisions. Furthermore, the substantial overlap between Early and Late FOVs and the adjusted *q*-values preclude definitive biological interpretation. At the FOV level, several morphology and chromatin-texture descriptors showed effect sizes, but none remained significant after multiple-comparison correction. Their biological interpretation is considered below. The findings can also be considered in the context of the rapidly expanding use of deep learning and transformer architectures for quantitative biomedical image analysis. Vision Transformers have demonstrated strong capacity for learning spatial representations across medical image classification and segmentation tasks [8,11,12], while quantitative cellular imaging studies have shown that nuclear morphology and chromatin organisation can provide informative representations of cellular state [2,23,24]. Previous work has further associated ageing and senescence with alterations in nuclear architecture, heterochromatin organisation, chromatin condensation, and higher-order genome structure [4,5,25,26]. The present results demonstrate heterogeneous time-associated phenotypic variation in DAPI-derived nuclear images, with moderate FOV-level effects across several morphology and chromatin-texture descriptors. Several morphology and chromatin-texture descriptors showed moderate effects, but none remained significant after FDR correction, while the primary AI models showed only modest discrimination under FOV-grouped validation. The stronger performance obtained for the widely separated timepoints suggests that learned representations may capture temporal nuclear variation, but the present study characterises this signal as time-associated nuclear phenotypic variation, providing a defined basis for subsequent molecular characterisation. The FOV-level distributions of the morphology and masked chromatin-texture descriptors are visualised in Figure 9.

### 4.1. Interpretation of Morphology and Chromatin Texture

The independent FOV-level analysis identified differences in nuclear morphology and chromatin texture across the experimental time course. Nuclear area showed the largest effect, with higher FOV-median values in the Late group (265.53 μm^2^) than in the Early group (219.05 μm^2^; Cohen’s d=0.591, permutation p=0.0406). Among the texture descriptors, GLCM correlation (d=−0.471), contrast (d=0.449), dissimilarity (d=0.414), and homogeneity (d=−0.355) showed moderate effects. However, none of the 12 candidate morphology and texture descriptors remained statistically significant after Benjamini–Hochberg correction (q≥0.442). These findings therefore represent candidate imaging associations rather than validated biomarkers. The observed texture differences warrant further investigation because previous studies have associated ageing and cellular senescence with changes in chromatin organisation, heterochromatin distribution, nuclear architecture, and lamina-associated domains [1,27,28,29]. Previous studies have also demonstrated that GLCM-based texture analysis can distinguish senescent from non-senescent cells, when evaluated alongside senescence-associated β-galactosidase staining [24], while quantitative chromatin-texture imaging has been used to differentiate proliferating, immortal, and senescent cellular states [23]. Ageing and senescence involve region-specific chromatin reorganisation, including loss or redistribution of heterochromatin and formation of condensed chromatin structures [25,26]. These observations provide biological motivation for investigating DAPI-derived texture features, but the correspondence between these previously reported mechanisms and the differences observed in the present dataset remains to be established. Importantly, the experimental groups in this study were defined by time in culture rather than molecularly validated ageing or senescence states. Consequently, the observed morphology and texture differences cannot be attributed specifically to nuclear biology because time in culture was confounded with experimental day and may also reflect day-associated variation in staining, cell density or confluence, illumination, focus, microscope configuration, sample handling, or other acquisition-related factors. Molecular validation is therefore required before these imaging features can be interpreted as ageing biomarkers.

The absence of FDR-significant individual descriptors is also relevant to the distinction between image features and validated imaging biomarkers. Quantitative imaging-biomarker frameworks emphasise that candidate features require reproducible extraction, appropriate statistical validation, and assessment across independent datasets or experimental conditions before translational interpretation [20,21]. From this perspective, the nominal association observed for nuclear area and the moderate effects observed for several GLCM descriptors should be regarded as prioritisation signals rather than evidence of established biomarkers. Their value lies in identifying specific nuclear characteristics that can now be tested prospectively against molecular reference measurements and independent biological replicates.

### 4.2. Translational Pathway Towards Imaging-Biomarker Validation

The present study provides a completed hierarchy-aware computational evaluation of candidate nuclear imaging signatures within the defined experimental dataset, representing the computational stage of imaging-biomarker development rather than validation of a diagnostic test. The subsequent stage of imaging-biomarker development is to evaluate the identified nuclear imaging signatures against independently measurable biological processes. Future experiments will therefore pair DAPI-derived morphology and chromatin-texture measurements with established markers of cellular ageing, senescence, DNA damage, and nuclear-lamina remodelling, including p16^INK4a^, p21, senescence-associated β-galactosidase, lamin B1, and γH2AX foci. Such measurements would provide independent biological reference standards against which the specificity of the imaging phenotype could be evaluated. The subsequent translational stage should examine whether molecularly supported nuclear imaging signatures reproduce across additional independent iPSC-CM cultures, experimental batches, and imaging sessions. Disease-specific or stress-induced cardiomyocyte models would then be required to determine whether the identified features discriminate biologically or pathologically meaningful cardiac phenotypes rather than general time-in-culture effects. Patient-derived iPSC-CMs could provide an intermediate translational platform for evaluating inter-individual heterogeneity and disease-specific phenotypes before clinical investigation. Ultimately, progression toward diagnostic or prognostic application would require validation in appropriately defined clinical or patient-derived cohorts with independent reference standards and prespecified endpoints. Such studies would need to evaluate diagnostic discrimination, calibration, reproducibility, sensitivity, specificity, and added value relative to established molecular or clinical measurements. Establishing these properties will require appropriately designed patient-derived or clinical validation studies. The present study instead provides a hierarchy-aware analytical framework for identifying candidate nuclear imaging signals that remain detectable across held-out source FOVs and can therefore be prioritised for subsequent evaluation through independent external biological replication.

### 4.3. Limitations

The findings should be interpreted within the experimental scope of the study. The analytical dataset comprised 154 quality-controlled nuclei from 51 source FOVs acquired across six experimental days and three independent culture batches. Source FOV was used as the grouping and primary evaluation unit because culture-batch identifiers were not encoded in the analytical dataset used for model development. The Early and Late groups represent operational time-in-culture categories rather than molecularly defined ageing or senescence states. Accordingly, the study evaluates time-associated nuclear imaging phenotypes and their cross-FOV reproducibility within the available experimental dataset. The secondary DINOv2 analysis was intentionally restricted to the more widely separated Days 1–2 and Days 5–6 to evaluate the influence of temporal separation. These findings establish a hierarchy-aware computational foundation for subsequent independent external biological replication and molecular evaluation of the identified nuclear imaging signatures.

## 5. Conclusions

The research presents a hierarchy-aware AI framework for analysing DAPI-derived nuclear phenotypes in human iPSC-derived cardiomyocytes while accounting for the hierarchical structure of microscopy data. The findings show that nuclear imaging differences can be detected across experimental timepoints, with stronger discrimination observed when the compared timepoints are more widely separated. The combined evaluation of deep learning, multiple-instance learning, morphology, chromatin texture, and attention-based analysis provides a structured approach for investigating time-associated nuclear phenotypes at the source-FOV level. The study further demonstrates the importance of preserving microscopy-field identity during model evaluation and statistical analysis to reduce information leakage and pseudoreplication. The proposed framework provides a rigorous preclinical strategy for identifying and prioritising candidate nuclear imaging signatures in iPSC-derived cardiomyocytes and supports their further investigation in biological and translational biomarker research.

## Figures and Tables

**Figure 1:**
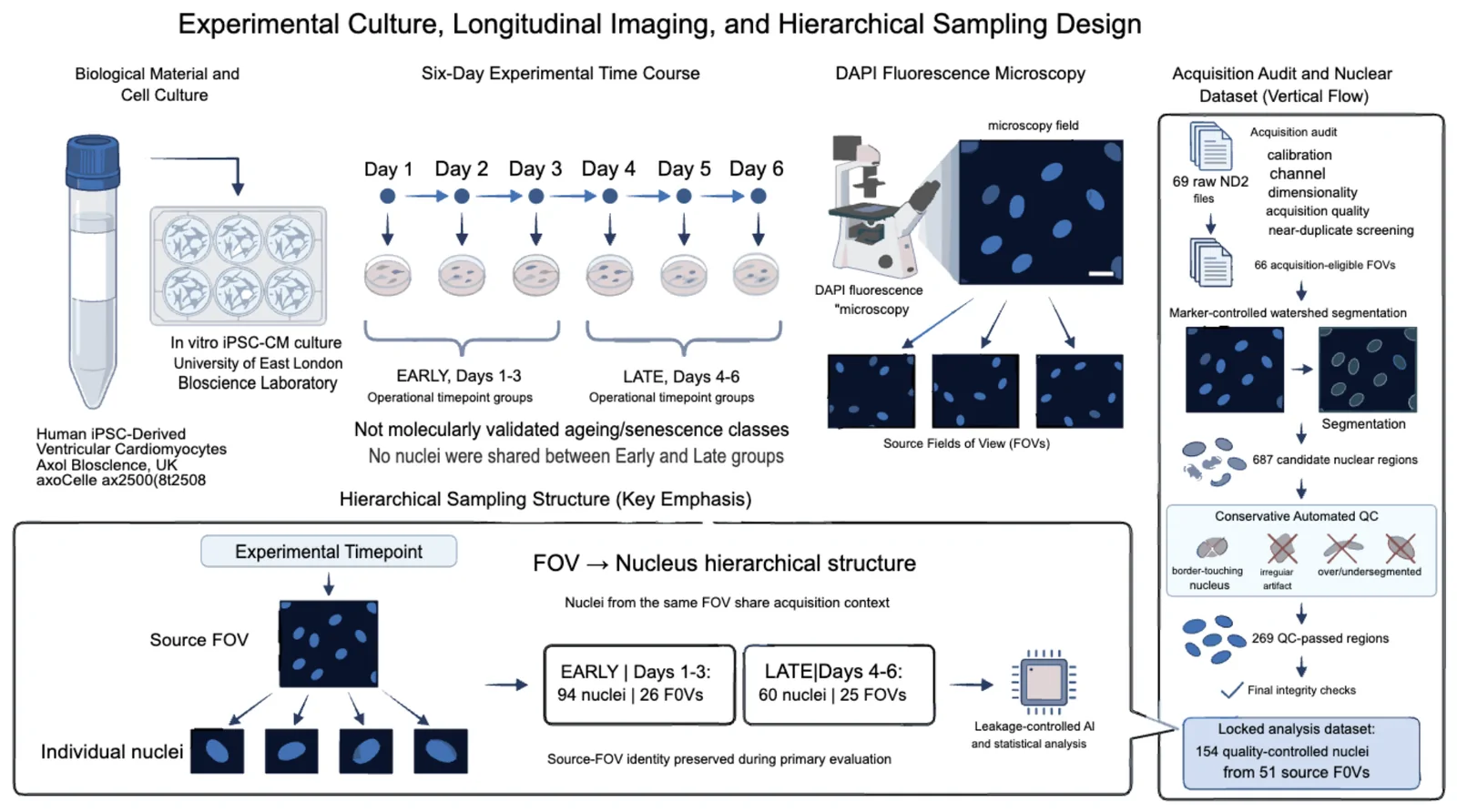
Experimental culture, time-course imaging, and hierarchical sampling design of the study. Human iPSC-derived ventricular cardiomyocytes were examined across a six-day experimental time course using DAPI fluorescence microscopy. Days 1–3 and Days 4–6 were operationally defined as Early and Late timepoint groups, respectively, and should not be interpreted as molecularly validated ageing or senescence classes. DAPI images were acquired from randomly selected FOVs during each imaging session rather than from predetermined or repeatedly imaged microscopic locations. Individual nuclei were therefore not longitudinally tracked across experimental days, and no individual nucleus was represented in both the Early and Late analytical groups. The acquisition archive contained 69 raw ND2 files, of which 66 FOVs remained eligible following calibration, channel, dimensionality, acquisition-quality, and near-duplicate screening. Marker-controlled watershed segmentation generated 687 candidate nuclear regions, with 269 regions passing conservative automated quality control. Final integrity checks produced the locked analytical dataset of 154 quality-controlled nuclei from 51 source FOVs, comprising 94 nuclei from 26 Early FOVs and 60 nuclei from 25 Late FOVs. Source-FOV identity was preserved throughout primary AI evaluation and statistical inference.

**Figure 2:**
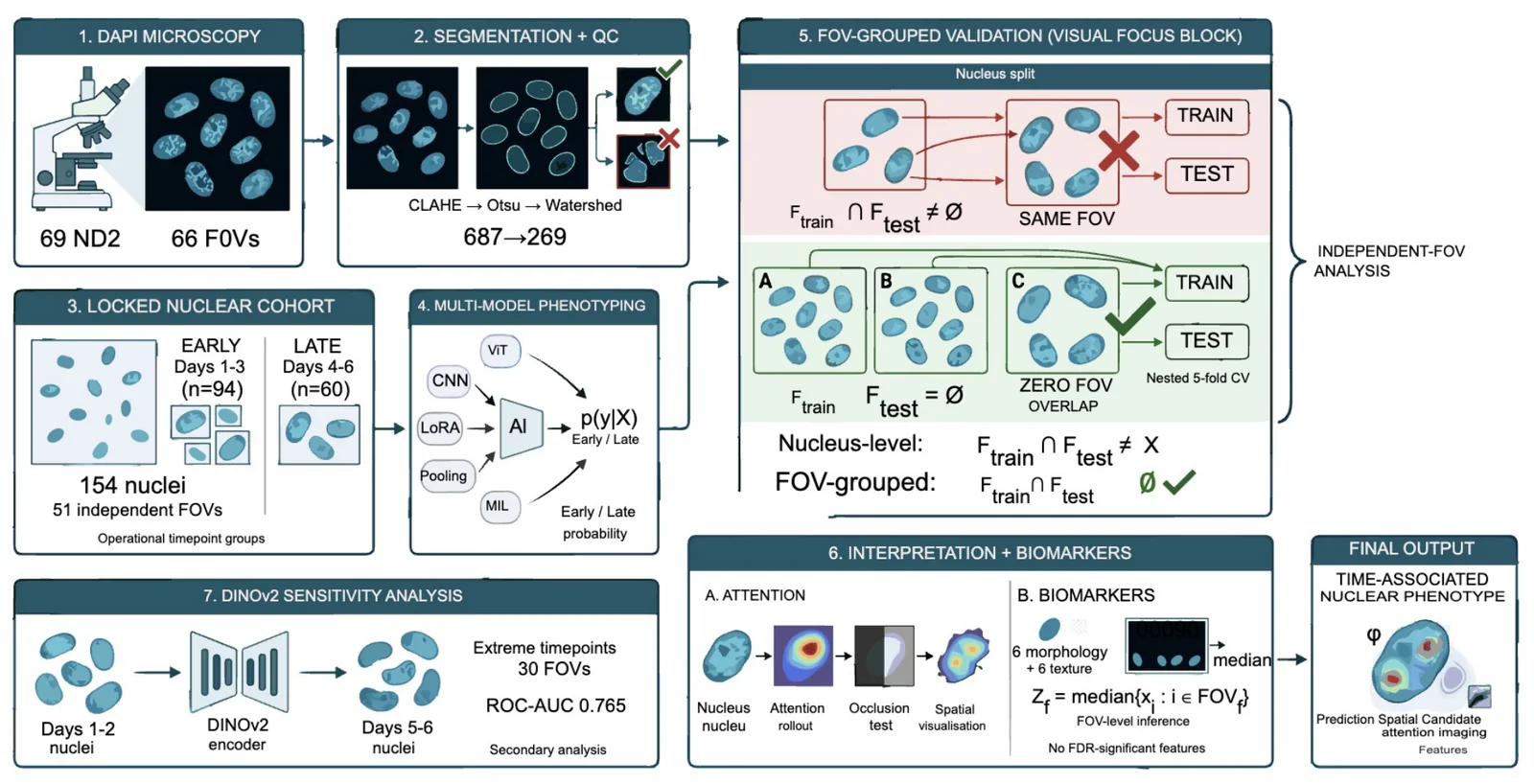
Leakage-controlled multi-model AI framework for time-associated nuclear phenotyping. DAPI fluorescence microscopy images were segmented and quality controlled to generate 154 nuclei ($X_{n}$) from 51 source FOVs used as the primary evaluation units, with operational Early and Late timepoint labels ($y_{n}$). Multiple AI models generated class predictions (${\hat{y}}_{n}$) under nested FOV-grouped cross-validation with zero train-test FOV overlap. Fold-matched attention rollout ($A_{\mathrm{rollout}}$) provided spatial model visualisation, while morphology and masked chromatin-texture descriptors were independently evaluated at the FOV level. A secondary DINOv2 analysis of extreme timepoints (Days 1–2 vs. Days 5–6; 30 FOVs) achieved a ROC-AUC of 0.765.

**Figure 3:**
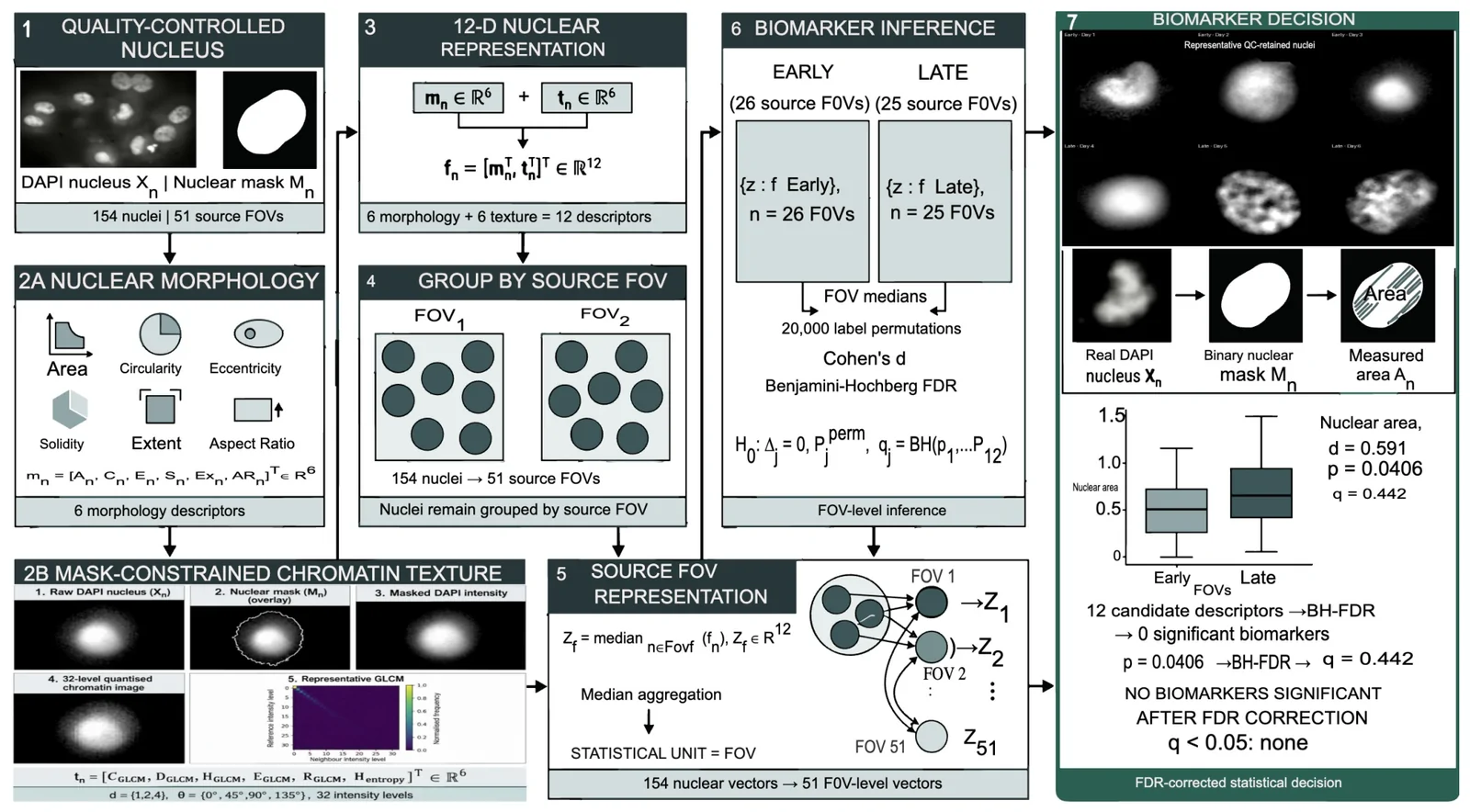
Nuclear biomarker extraction and FOV-level statistical inference framework. From 154 quality-controlled nuclei, six morphological descriptors ($m_{n}\in R^{6}$) and six mask-constrained chromatin-texture descriptors ($t_{n}\in R^{6}$) were concatenated into a 12-dimensional nuclear representation ($f_{n}\in R^{12}$). Nucleus-level vectors were grouped by source FOV and median-aggregated into 51 FOV-level representations, comprising 26 Early and 25 Late FOVs. Biomarker inference used 20,000 label permutations, Cohen’s *d*, and Benjamini–Hochberg false-discovery-rate (BH-FDR) correction across the 12 candidate descriptors. Nuclear area showed the strongest nominal association (d=0.591, p=0.0406), but did not remain significant after multiple-testing correction (q=0.442); consequently, none of the candidate descriptors satisfied the prespecified FDR significance criterion of q<0.05.

**Figure 4:**
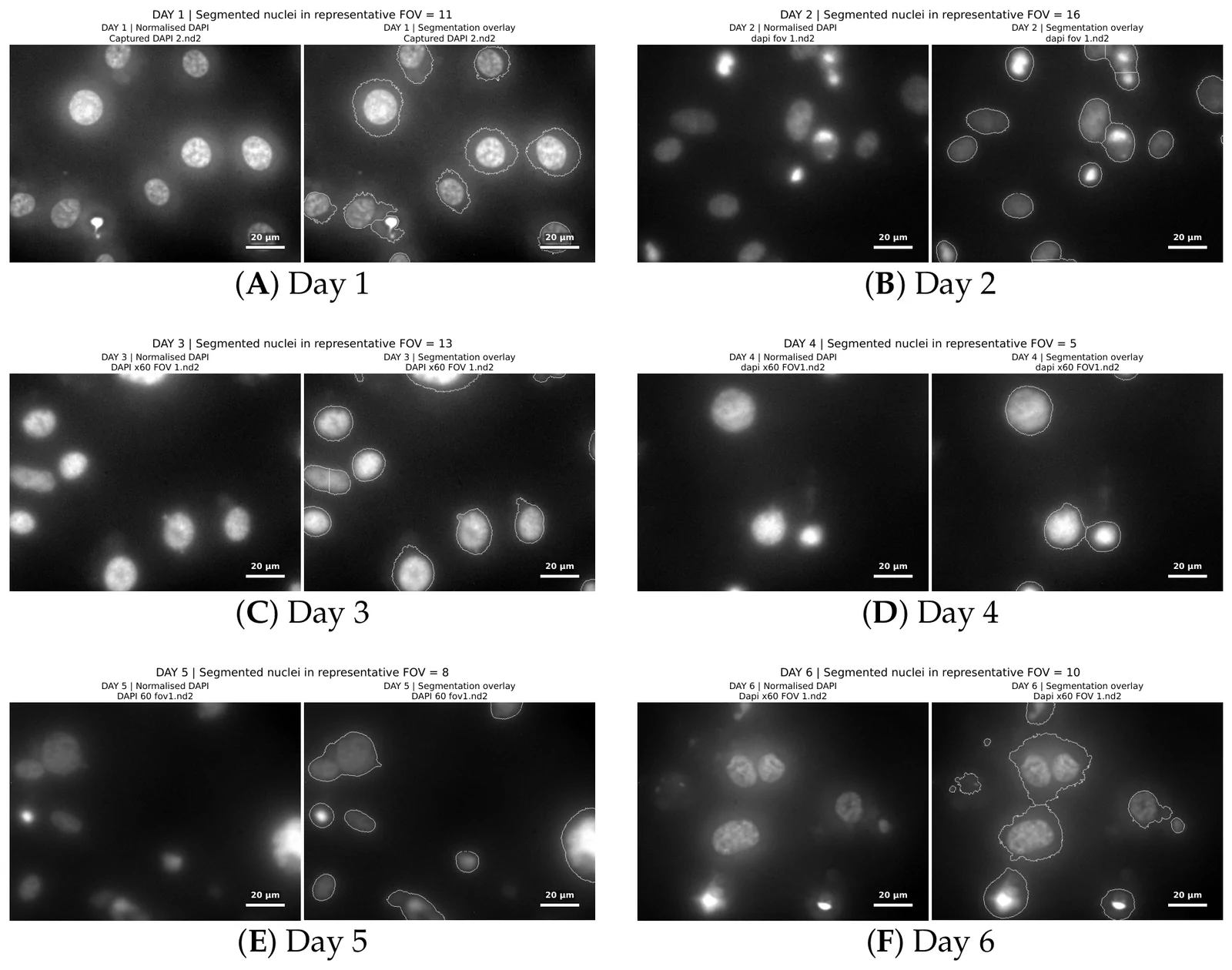
Representative visualisation of the marker-controlled watershed nuclear-segmentation pipeline across the six-day experimental time course. For each experimental day, the left image shows the normalised DAPI fluorescence microscopy field and the right image shows the corresponding segmentation-boundary overlay on the same normalised field. The same normalisation procedure and intensity-display limits were applied to all representative DAPI images. Scale bars represent 20 μm. The numbers displayed within the individual panels indicate the segmented nuclei detected in the corresponding representative source FOV: 11 for Day 1, 16 for Day 2, 13 for Day 3, 5 for Day 4, 8 for Day 5, and 10 for Day 6. These values are representative-FOV segmentation outputs rather than dataset-level nuclear counts. The panels illustrate the behaviour of the segmentation pipeline across fields with variation in nuclear appearance and density. Downstream analytical inclusion was determined using the automated segmentation and morphometric quality-control procedure together with the blinded visual audit described in the Methods.

**Figure 5:**
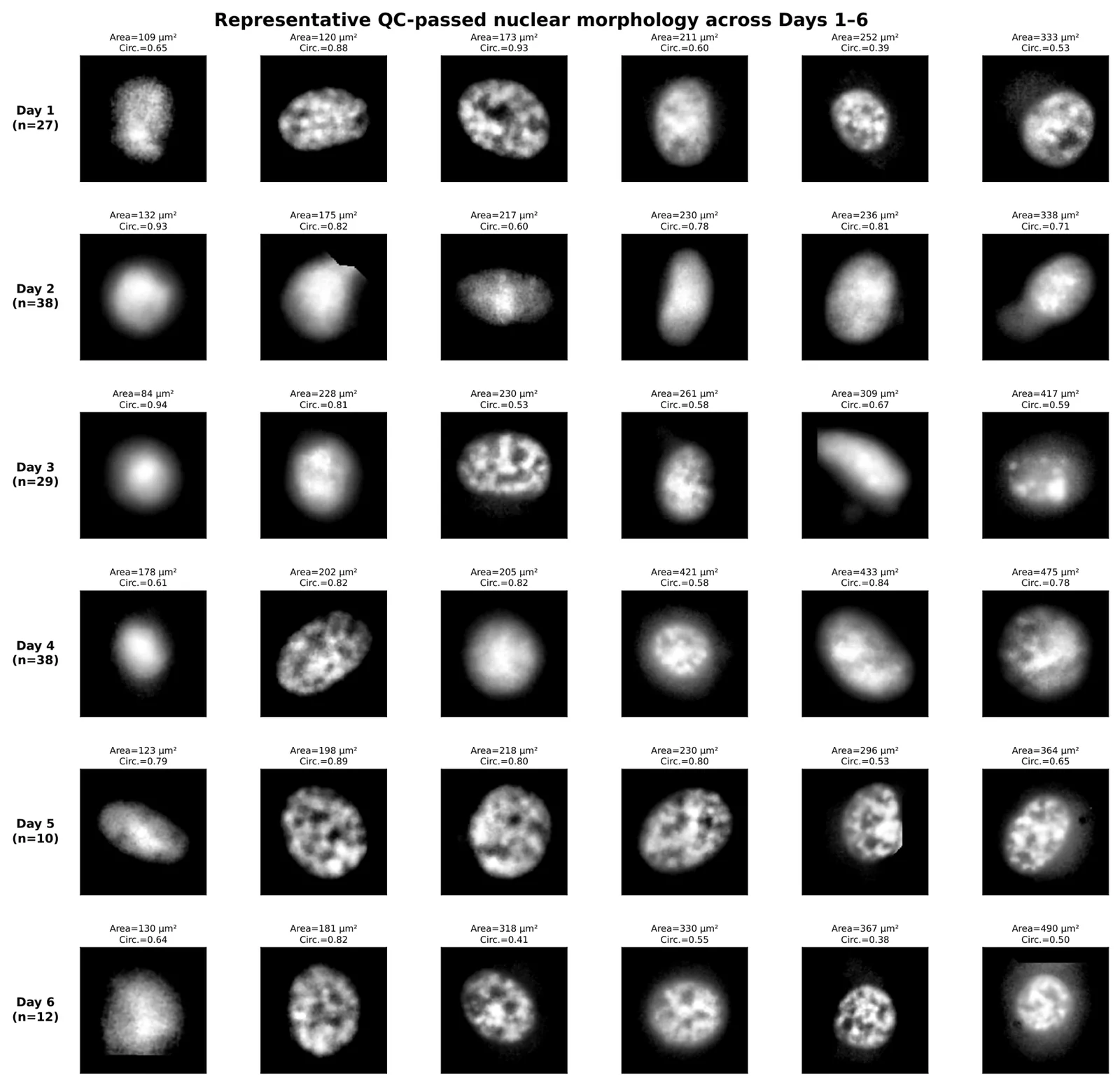
Representative quality-controlled nuclear morphology across the six-day experimental time course. Six nuclei are displayed for each day from the final locked analytical dataset, which contained 27, 38, 29, 38, 10, and 12 analytical nuclei for Days 1–6, respectively. The displayed examples were selected deterministically using the six established morphological descriptors: nuclear area, circularity, eccentricity, solidity, extent, and aspect ratio with each displayed nucleus within a given day originating from a different source FOV. Nuclear area and circularity are reported above each crop. Among the displayed examples, Day 1 nuclear area ranged from 109–333 μm^2^ with circularity 0.39–0.93; Day 2 from 132–338 μm^2^ with circularity 0.60–0.93; Day 3 from 84–417 μm^2^ with circularity 0.53–0.94; Day 4 from 178–475 μm^2^ with circularity 0.58–0.84; Day 5 from 123–364 μm^2^ with circularity 0.53–0.89; and Day 6 from 130–490 μm^2^ with circularity 0.38–0.82. Across all 36 displayed nuclei, area ranged from 84–490 μm^2^ and circularity from 0.38–0.94, illustrating the range of nuclear size and shape represented by the selected examples. The values in parentheses beside each day denote the total number of nuclei in the final locked analytical dataset for that day, whereas six representative nuclei are displayed. The displayed nuclei are intended to illustrate morphological heterogeneity and should not be interpreted as independent biological replicates or as estimates of the complete day-specific morphological distributions.

**Figure 6:**
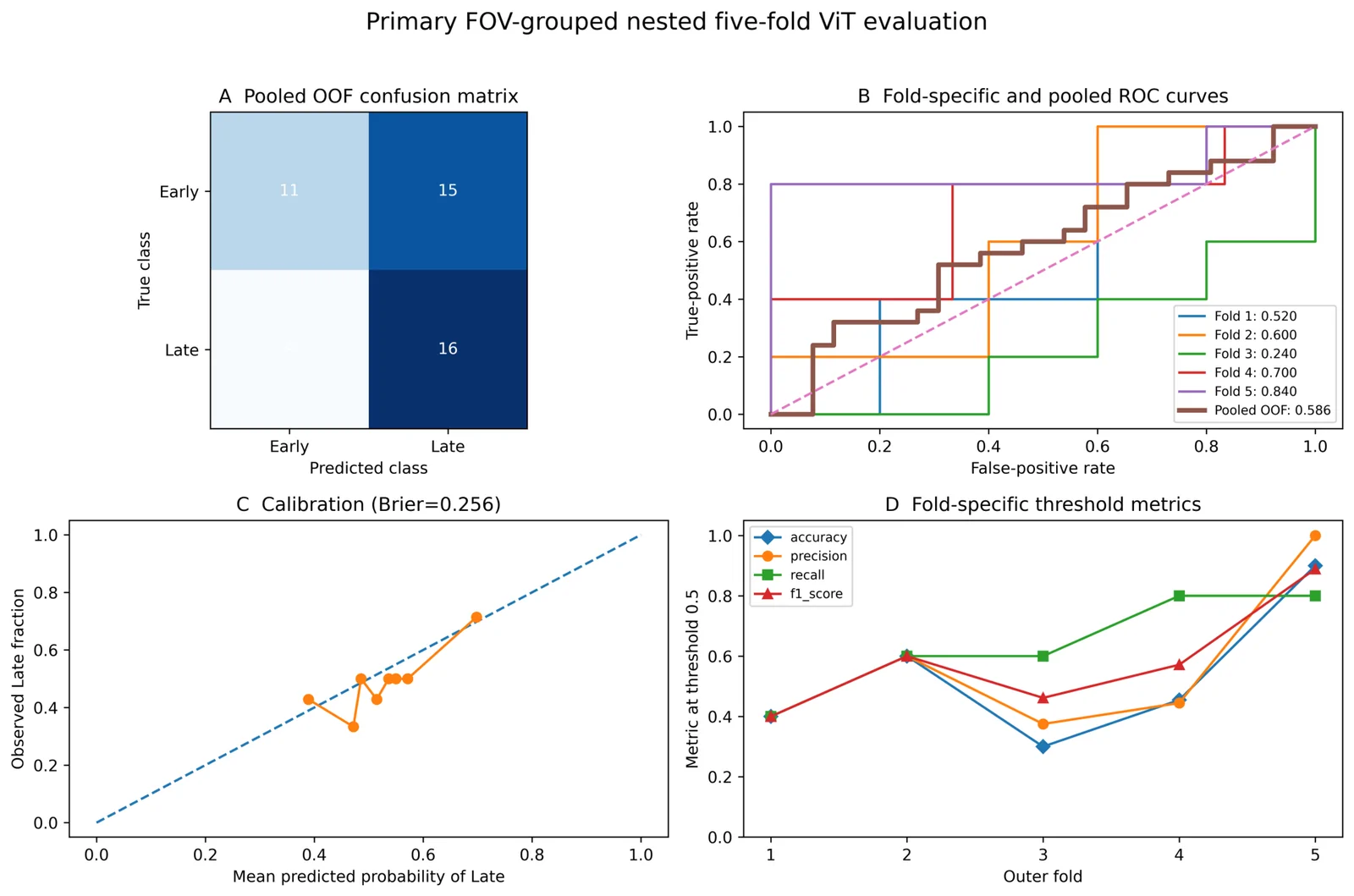
Primary FOV-grouped nested five-fold evaluation of the ViT-B/16 model under the leakage-controlled evaluation protocol. (**A**) Pooled out-of-fold confusion matrix obtained by combining predictions from all held-out FOVs across the five outer folds. The model correctly classified 11 of 26 Early FOVs and 16 of 25 Late FOVs, demonstrating higher sensitivity for the Late class. (**B**) Receiver operating characteristic (ROC) curves for each outer fold together with the pooled out-of-fold ROC curve. Fold-specific ROC-AUC values ranged from 0.240 to 0.840, while the pooled out-of-fold ROC-AUC was 0.586, illustrating substantial variability between folds but modest pooled discrimination. (**C**) Calibration plot comparing predicted Late-class probabilities with the observed Late-class frequencies. (**D**) Fold-specific threshold-dependent performance metrics at the default decision threshold of 0.5. Accuracy, precision, recall, and F1-score varied considerably across the five outer folds, reflecting the limited number of independent microscopy fields and the challenging nature of the Early-versus-Late classification task.

**Figure 7:**
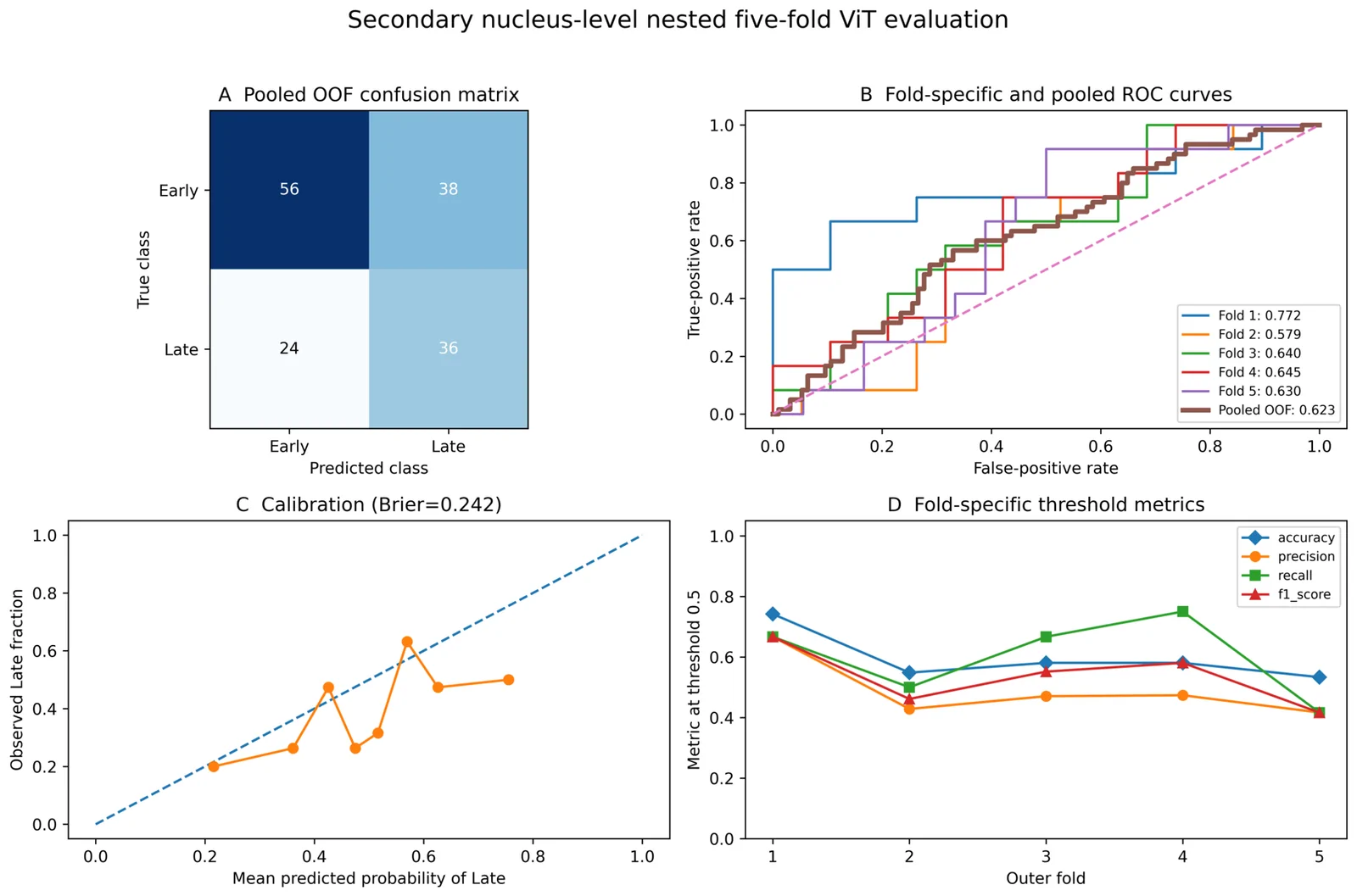
Secondary nucleus-level nested five-fold evaluation of the ViT-B/16 model. (**A**) The pooled out-of-fold confusion matrix shows 56 correctly classified Early nuclei, 38 Early nuclei classified as Late, 36 correctly classified Late nuclei, and 24 Late nuclei classified as Early. (**B**) Fold-specific receiver operating characteristic curves and the pooled out-of-fold ROC curve, with a pooled ROC-AUC of 0.623. (**C**) Calibration analysis yielded a Brier score of 0.242; the dashed diagonal represents perfect calibration. (**D**) Fold-specific accuracy, precision, recall, and F1-score at the fixed decision threshold of 0.5. This nucleus-level analysis is reported as a secondary sensitivity analysis because nuclei originating from the same source FOV are not statistically independent.

**Figure 8:**
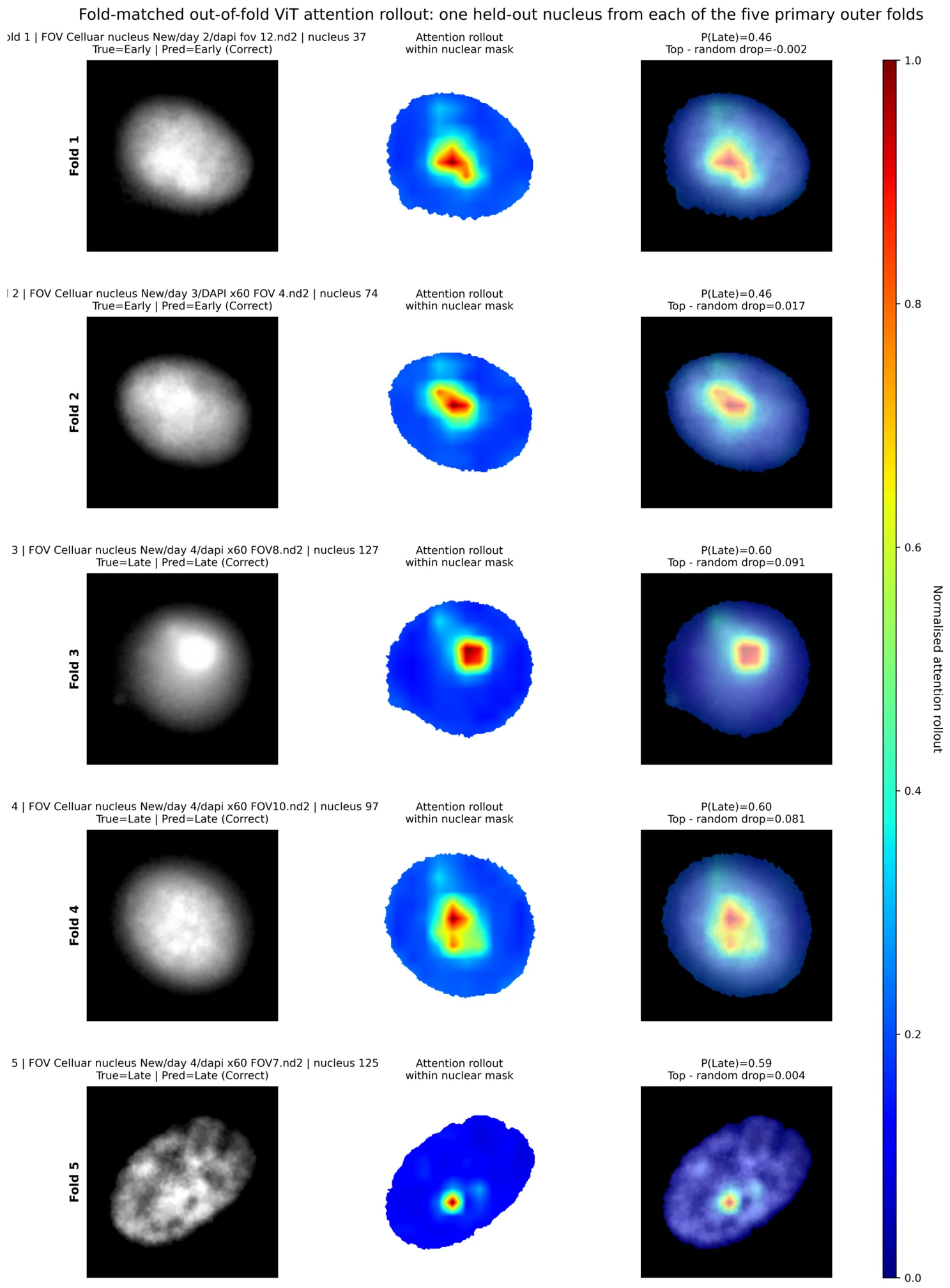
Fold-matched out-of-fold ViT attention rollout across five primary FOV-grouped outer folds. Each row shows a representative held-out nucleus, mask-constrained normalised attention rollout, and overlay generated using the corresponding fold-specific seed-42 ViT-B/16 checkpoint. Top-minus-random drop quantifies the occlusion-based faithfulness assessment. The attention maps are presented as spatial visualisations only; FOV-level testing did not provide statistically significant evidence of attention faithfulness.

**Figure 9:**
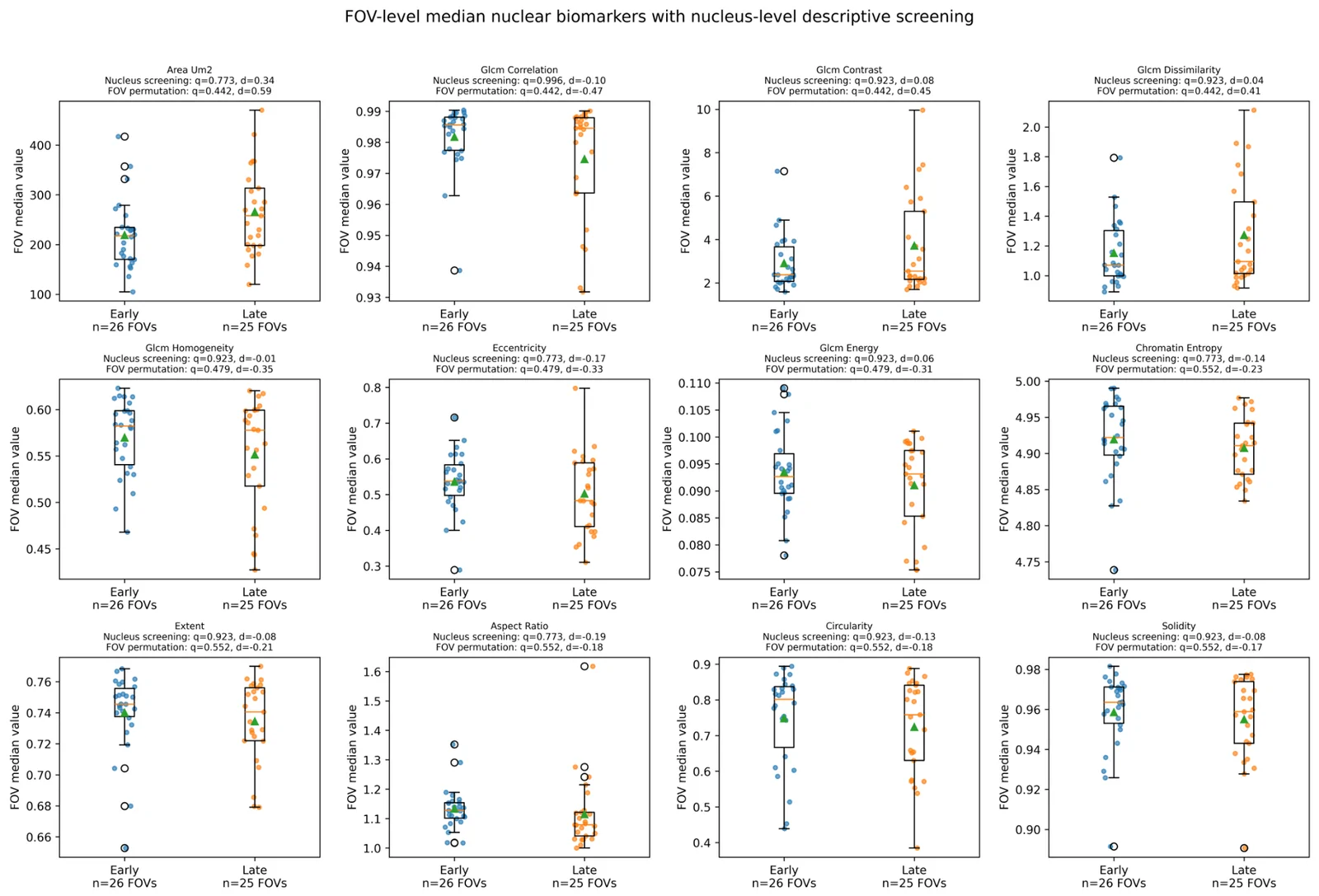
Independent FOV-level comparison of six morphology and six masked chromatin-texture descriptors. Each point represents the median value for one source FOV, with 26 Early and 25 Late FOVs. The panels report nucleus-level candidate biomarker screening and primary FOV-level permutation results.

**Table 1:** Principal scientific contributions of this study.

Contribution	Description
Novel longitudinal microscopy dataset	A novel laboratory-generated time-course DAPI fluorescence microscopy dataset of human iPSC-derived cardiomyocytes acquired across six sequential experimental timepoints under controlled conditions, with source-FOV provenance preserved throughout image processing, nuclear segmentation, quality control, AI modelling, and statistical analysis.
Primary prediction task	Binary classification of operationally defined Early (Days 1–3) versus Late (Days 4–6) time-in-culture groups from DAPI-derived nuclear images, with model performance evaluated at the source-FOV level under leakage-controlled FOV-grouped cross-validation.
Hierarchy-aware validation	A nested five-fold FOV-grouped evaluation framework in which all nuclei from the same microscopy field remained within one fold, with inner grouped model selection, three training seeds, pooled out-of-fold prediction, and FOV-level bootstrap confidence intervals.
Multi-model comparison	A matched comparison of ViT-B/16, ResNet50, EfficientNet-B0, ConvNeXt-Tiny, LoRA-adapted ViT, frozen-ViT neural pooling, attention-based multiple-instance learning, morphology-aware gated-attention MIL, and handcrafted morphology and texture classifiers.
Self-supervised sensitivity analysis	A DINOv2-based multi-view ensemble combining self-supervised image embeddings, frozen ViT representations, morphology, and chromatin texture for discrimination of clearly separated experimental timepoints.
Quantitative imaging analysis	FOV-level evaluation of six morphology and six masked chromatin-texture descriptors using median aggregation, class-label permutation testing, effect-size estimation, and Benjamini–Hochberg correction.
Interpretability audit	Fold-matched out-of-fold attention rollout combined with attention-targeted and random patch occlusion, explicitly distinguishing spatial visualisation from validated explanation faithfulness.
Translational biomarker framework	A preclinical evaluation strategy for determining whether DAPI-derived nuclear imaging signatures remain detectable across held-out source microscopy fields before progression to molecular validation, disease-specific modelling, independent external biological replication, and future translational evaluation.

**Table 2:** Cell-culture, microscopy acquisition, and experimental sampling characteristics of the dataset.

Experimental Component	Study Configuration
Cell model	Commercially available human induced pluripotent stem cell-derived ventricular cardiomyocytes (axoCells™, Catalogue No. ax2500/ax2508; Axol Bioscience, UK).
Culture format	Cardiomyocytes were cultured in 96-well plates, with an approximate growth area of 0.34 cm^2^ per well.
Cell preparation and seeding	Approximately 700,000–1,000,000 cardiomyocytes were available following thawing and revival, with approximately 34,000–68,000 cells seeded per well depending on the experimental preparation.
Culture conditions	Cardiomyocytes were maintained at 37 °C, 5% CO_2_, and approximately 20% O_2_ under controlled incubator conditions.
Independent biological replication	The experiments comprised three independent culture batches, which constituted the biological replicates. DAPI images were acquired across all three independent cultures, with multiple randomly selected FOVs providing within-culture image sampling. Source FOVs therefore represent imaging-level sampling units rather than independent biological replicates.
Well-level sampling	The experimental platform used 96-well plates; however, the exact number of individual wells contributing to the 69 raw DAPI ND2 acquisitions was not recorded in the analytical dataset used for this study. Consequently, source FOV rather than well was the highest identifiable grouping unit available for leakage-controlled analysis.
Experimental observation period	Cardiomyocytes were monitored longitudinally across sequential experimental days. The DAPI nuclear phenotyping analysis reported in this study used Days 1–6.
Longitudinal sampling unit	DAPI images were acquired from randomly selected FOVs during each imaging session. FOVs were not selected based on previously imaged locations and were not intended to represent repeated imaging of the same microscopic locations, wells, or nuclei across experimental days. Accordingly, the DAPI dataset represents repeated sampling across experimental timepoints rather than longitudinal tracking of individual nuclei or microscopic regions.
Imaging laboratory	University of East London Bioscience Laboratory.
Microscopy system	A Nikon Eclipse Ti2 microscope (Nikon, Singapore) was used for high-resolution cardiomyocyte imaging. An EVOS XL imaging system was additionally used for routine sample visualisation.
Objective	A 60× oil-immersion objective was used for high-resolution imaging.
Imaging schedule	Imaging was performed daily at approximately 09:00 h (±30 min) to maintain temporal consistency across the longitudinal experiment.
Image normalisation	DAPI fluorescence images were processed using a consistent preprocessing pipeline across all experimental days, including CLAHE-based contrast enhancement and intensity normalisation before segmentation and quantitative analysis. This normalisation was applied uniformly across the dataset to reduce variation in image intensity and contrast between acquisitions.
Day-associated technical variation	The available acquisition documentation did not establish that staining, microscope settings, illumination, focus, cell density or confluence, and sample-handling conditions were randomised across experimental days. Consequently, potential day-associated technical variation cannot be separated from time-in-culture effects in the present dataset.
FOV acquisition	Approximately 5–10 FOVs per well were acquired depending on cell density. Across the broader acquisition protocol, approximately 5–15 images/videos were collected per experimental day.
FOV inclusion criteria	Fields were retained when the cellular region was adequately in focus and contained sufficient cardiomyocyte material for quantitative analysis.
FOV exclusion criteria	Fields affected by substantial cell overlap, debris, bubbles, motion blur, or other visible acquisition artefacts were excluded from downstream analysis.
Sterile handling	Cell handling and sample preparation were performed under biosafety hoods #4 and #5 using sterile laboratory procedures.
DAPI imaging	Nuclei were visualised using DAPI fluorescence microscopy for quantitative assessment of nuclear morphology and chromatin organisation.
Raw DAPI acquisition archive	The DAPI acquisition archive contained 69 raw ND2 microscopy files/FOVs.
Acquisition-level screening	Calibration, fluorescence-channel, dimensionality, acquisition-quality, and near-duplicate checks were performed before nuclear segmentation.
Acquisition-eligible FOVs	Following acquisition-level screening, 66 FOVs were retained for image processing.
Segmentation	Nuclei were segmented from CLAHE-enhanced images using Otsu thresholding, morphological refinement, distance transformation, and marker-controlled watershed segmentation.
Candidate nuclear regions	Marker-controlled watershed segmentation generated 687 candidate nuclear regions.
Region-level quality control	Of the 687 segmented candidate nuclear regions, 269 (39.2%) passed automated morphometric and segmentation-quality screening. The automated criteria included physical nuclear area, circularity, eccentricity, solidity, extent, source-border proximity, straight or truncated boundaries, and shape-based safeguards against merged nuclei, attached fragments, and segmentation artefacts. Day- and group-specific QC outcomes are reported; retention was 40.1% for the Early group and 37.9% for the Late group. The QC thresholds were informed by segmentation and morphometric quality diagnostics, fixed before downstream modelling, and were not selected or adjusted using Early/Late group differences, class labels, or downstream classification performance.
QC selection considerations	The 39.2% automated QC retention rate reflects conservative exclusion of segmented regions that did not satisfy the fixed nuclear morphology and segmentation-integrity criteria rather than selection according to Early/Late labels or model predictions. Because QC can preferentially retain nuclei with particular geometric or image-quality characteristics, some influence of QC selection on the distribution of retained nuclear phenotypes cannot be completely excluded. To reduce outcome-driven selection, QC was performed before AI modelling and the automated thresholds were not optimised against classification accuracy or other model-performance measures.
Final analytical dataset	Following final dataset-integrity checks, 154 quality-controlled nuclei from 51 source FOVs were retained for the locked analytical dataset.
Timepoint definition	Days 1–3 were operationally defined as the Early time-in-culture group and Days 4–6 as the Late time-in-culture group. These labels represent relative time in culture and do not denote molecularly validated ageing, maturation, senescence, or disease states.
Timepoint distribution	The locked dataset contained 94 Early nuclei from 26 source FOVs and 60 Late nuclei from 25 source FOVs.
Analytical hierarchy	The experiments comprised three independent culture batches, which constituted the biological replicates, with randomly sampled source FOVs providing within-culture image sampling at each experimental timepoint and nuclei nested within source FOVs. Individual nuclei originating from the same source FOV were not treated as independent evaluation units.
Primary evaluation unit	Source FOV was used as the primary grouping and evaluation unit. Source-FOV identity was preserved during cross-validation, statistical inference, and uncertainty estimation, ensuring that nuclei from the same FOV did not appear in both training and test partitions.
Higher-level independence	FOV-grouped analysis addresses dependence among nuclei sharing the same microscopy field. The experiments comprised three independent culture batches as biological replicates, with multiple randomly sampled FOVs providing within-culture image sampling. Because culture-batch identifiers were not encoded in the analytical dataset used for model development, the computational evaluation remained grouped at the source-FOV level.

**Table 3:** Automated quality-control pipeline for nucleus selection prior to Vision Transformer training.

Component	Configuration
Input images	DAPI fluorescence images extracted from acquisition-eligible ND2 files
Image preprocessing	CLAHE contrast enhancement followed by intensity normalisation
Segmentation method	Otsu thresholding, morphological refinement, distance transform and marker-controlled watershed
Coarse minimum object area	300 pixels
Coarse maximum object area	15% of the source-image area, used as an upper safeguard against implausibly large segmented regions
Calibrated nuclear-area range	80–490 μm^2^ for region-level nuclear QC
Minimum circularity	0.35
Maximum eccentricity	0.95
Minimum solidity	0.85
Extent range	0.30–0.95
Maximum straight-edge fraction	<0.20
Source-border exclusion	Objects touching the source-image boundary were excluded
Near-border exclusion	Objects within 10 pixels of the source-image boundary were excluded
Crop edge exclusion	Crops intersecting image borders were removed
Crop padding	24 pixels around each segmented nucleus
Crop size	Resized to 224×224 pixels
Composite quality score	Composite score combining circularity (35%), solidity (25%), inverse eccentricity (20%) and fill ratio (20%). Fill ratio contributed to this composite score but was not applied as a separate hard QC exclusion threshold.
Blinded visual QC audit	Visual review was performed using randomised object identifiers without access to experimental day, time-in-culture group, source-FOV identity, quantitative morphology measurements, and model outputs.
Region-level QC outcome	269 of 687 segmented candidate regions passed conservative automated QC (39.2%)
FOV-level QC screen	Median of 4 QC-passed candidates per FOV; no over-quantity outlier FOVs; seven FOVs contained no QC-passed candidates

**Table 4:** Canonical deep-learning and transfer-learning configuration.

Component	Configuration
Prediction task	Binary classification of Early (Days 1–3) and Late (Days 4–6) timepoint groups
Input image	224×224 quality-controlled grayscale nuclear crop replicated to three channels
Pretrained architectures	ViT-B/16 (ImageNet-1K), ResNet50 (ImageNet-1K), EfficientNet-B0 (ImageNet-1K), and ConvNeXt-Tiny (ImageNet-1K)
Initial warm-up	Three epochs with all pretrained backbone weights frozen and only the binary classification head trainable
ViT-B/16 fine-tuning	Final transformer encoder block and classification head trainable; all preceding pretrained encoder blocks frozen
ResNet50 fine-tuning	Layer4 and classification head trainable; earlier pretrained backbone layers frozen
EfficientNet-B0 fine-tuning	Final two feature modules and classification head trainable; remaining pretrained feature layers frozen
ConvNeXt-Tiny fine-tuning	Final two feature modules and classification head trainable; remaining pretrained feature layers frozen
LoRA ViT-B/16	Pretrained ViT weights remained frozen; LoRA parameters were applied to the fused query-key-value attention projection of the final four transformer blocks and trained together with the classification head. LoRA rank r=4, α=8, LoRA dropout =0, and classification-head dropout =0.20
Frozen-ViT models	Pretrained ViT backbone used as a fixed feature extractor without end-to-end backbone fine-tuning; downstream FOV-level pooling and classification components were trained separately
DINOv2 sensitivity analysis	Pretrained DINOv2 ViT-S/14 used as a frozen feature extractor with no backbone fine-tuning; DINOv2 representations were combined with frozen ViT, morphology, and masked chromatin-texture representations for the secondary Days 1–2 versus Days 5–6 analysis
Outer validation	Five-fold class-stratified FOV-grouped cross-validation with zero FOV overlap
Inner validation	Class-balanced grouped inner training/validation split within each outer training fold
Random seeds	42, 7, and 21
Loss	Class-weighted cross-entropy with label smoothing of 0.05
Optimiser and learning rates	AdamW; backbone/head learning rates: ViT-B/16, $1\times 10^{-5}/1\times 10^{-4}$; ResNet50, $1\times 10^{-4}/5\times 10^{-4}$; EfficientNet-B0, $1\times 10^{-4}/5\times 10^{-4}$; ConvNeXt-Tiny, $5\times 10^{-5}/2\times 10^{-4}$
Weight decay and gradient clipping	Weight decay $=5\times 10^{-4}$; maximum gradient norm =1.0
Batch size	8
Maximum fine-tuning epochs	40
Early stopping	Patience of eight epochs using inner validation loss
Decision threshold	0.5 for primary reporting
Uncertainty	5000 stratified FOV-level bootstrap resamples
Implementation	Python 3.12, PyTorch 2.6, TorchVision 0.21, Scikit-learn 1.6, NumPy 2.0.2, SciPy 1.16.3, and scikit-image 0.25.2

**Table 5:** Locked dataset and evaluation units.

Metric	Nuclei	FOVs
Early, Days 1–3	94	26
Late, Days 4–6	60	25
Total	154	51

**Table 6:** Model validation and statistical evaluation hierarchy.

Component	Configuration
Primary validation	Nested five-fold FOV-grouped cross-validation
Secondary nucleus-level sensitivity benchmark	Nested nucleus-stratified five-fold cross-validation without source-FOV grouping; reported only as a sensitivity benchmark
Repeated training	Three random seeds: 42, 7, and 21
Outer-fold integrity	Every nucleus assigned to one outer test fold; zero primary FOV overlap
Primary evaluation unit	Source FOV
Primary metrics	Accuracy, precision, recall, F1-score, ROC-AUC, average precision, and Brier score
Confidence intervals	5000 stratified FOV-level bootstrap resamples
Primary deep-learning model	ViT-B/16
Secondary deep-learning comparisons	ResNet50, EfficientNet-B0, and ConvNeXt-Tiny
Secondary modelling analyses	LoRA ViT, frozen-ViT neural pooling, attention MIL, and hybrid morphology-aware gated-attention MIL
Self-supervised sensitivity analysis	DINOv2 multi-view FOV ensemble for Days 1–2 versus Days 5–6
Handcrafted ablation	Morphology-only, masked texture-only, and morphology plus masked texture
Biomarker inference	FOV-median aggregation and two-sided class-label permutation testing
Multiplicity correction	Benjamini–Hochberg false discovery rate
Explainability	Fold-matched attention rollout and attention-versus-random occlusion

**Table 7:** Quality-controlled nuclear dataset across experimental days.

Day	Accepted Nuclei	Source FOVs	Timepoint Group
Day 1	27	7	Early
Day 2	38	10	Early
Day 3	29	9	Early
Day 4	38	12	Late
Day 5	10	6	Late
Day 6	12	7	Late
Total	154	51	-

**Table 8:** Leading associations from the FOV-level biomarker analysis. All adjusted *q*-values exceeded the prespecified significance threshold after Benjamini–Hochberg correction across 12 comparisons.

Biomarker	Type	Late-Early Difference	Cohen’s *d*	Permutation *p*	Adjusted *q*
Nuclear area (μm^2^)	Morphology	46.478	0.591	0.0406	0.442
GLCM correlation	Texture	−0.00718	−0.471	0.0991	0.442
GLCM contrast	Texture	0.809	0.449	0.1180	0.442
GLCM dissimilarity	Texture	0.121	0.414	0.1473	0.442
GLCM homogeneity	Texture	−0.0184	−0.355	0.2138	0.479

**Table 9:** Pooled out-of-fold performance of deep-learning architectures under the primary FOV-grouped nested five-fold protocol. All models were evaluated on the same 51 source FOVs under the FOV-grouped protocol.

Model	Accuracy	Precision	Recall	F1-Score	ROC-AUC	Average Precision	Brier Score
ViT-B/16	0.529	0.516	0.640	0.571	**0.586**	**0.570**	**0.256**
ResNet50	0.529	0.526	0.400	0.455	0.477	0.513	0.258
EfficientNet-B0	0.471	0.467	0.560	0.509	0.429	0.470	0.265
ConvNeXt-Tiny	**0.569**	**0.552**	**0.640**	**0.593**	0.543	0.562	0.269

**Table 10:** Pooled out-of-fold performance of handcrafted feature classifiers under the primary FOV-grouped nested five-fold protocol.

Feature Set	Accuracy	Precision	Recall	F1-Score	ROC-AUC	Average Precision	Brier Score
Morphology-only	0.529	0.516	0.640	0.571	0.535	0.555	0.252
Masked texture-only	0.471	0.455	0.400	0.426	0.380	0.423	0.259
Morphology + masked texture	0.471	0.462	0.480	0.471	0.457	0.488	0.261

**Table 11:** Primary pooled out-of-fold ViT-B/16 performance with FOV-level bootstrap confidence intervals.

		95% Bootstrap CI	
Metric	Point Estimate	Lower	Upper
Accuracy	0.529	0.392	0.667
Precision	0.516	0.400	0.630
Recall	0.640	0.440	0.800
F1-score	0.571	0.429	0.692
ROC-AUC	0.586	0.420	0.743
Average precision	0.570	0.463	0.762
Brier score ^a^	0.256	0.229	0.287

Pooled out-of-fold predictions from nested five-fold FOV-grouped cross-validation over 51 source fields of view (seeds 42, 7, 21). Confidence intervals were estimated from B=5000 stratified bootstrap resamples of the pooled held-out source-FOV predictions after averaging predicted probabilities across the three training seeds. The complete model-training and nested cross-validation pipeline was not repeated within individual bootstrap resamples; the intervals therefore quantify FOV-sampling uncertainty conditional on the fitted evaluation procedure. Threshold-dependent metrics used a decision threshold of 0.5. ^a^ Lower values indicate better probabilistic prediction. The Brier score was used as an evaluation metric and was not used for model optimisation, early stopping, or model selection.

**Table 12:** Locked attention-texture coupling analysis using all 154 nuclei and 51 source FOVs. Nucleus-level correlations characterise within-nucleus attention-texture associations, while primary statistical inference was conducted at the FOV level to account for dependence among nuclei originating from the same source FOV. Cohen’s *d* represents the standardized Late-versus-Early difference in FOV-level coupling and was interpreted together with the permutation test and BH-adjusted *q*-value.

Measure	Result
Locked nuclei analysed	154/154
Source FOVs	51
Nucleus-level mean Spearman ρ	−0.0545
Nucleus-level median Spearman ρ	−0.0940
Mean FOV-median observed ρ	−0.0098
Mean FOV-median shuffled-null ρ	0.0008
Observed-minus-null difference	−0.0106
Bootstrap 95% CI for observed-minus-null	−0.0810 to 0.0654
Observed versus shuffled-null Wilcoxon *p*	0.5486
Early mean FOV-median ρ	−0.0670
Late mean FOV-median ρ	0.0497
Late-minus-Early difference	0.1166
Cohen’s *d*	0.446
Early-versus-Late permutation *p*	0.1178
BH-adjusted *q* for Early-versus-Late comparison	0.2357

**Table 13:** Comparison of secondary nucleus-level and primary FOV-grouped analyses. The nucleus-level analysis evaluated discrimination among individual nuclei, whereas the FOV-grouped analysis evaluated cross-field generalisation without source-FOV overlap between training and test sets.

Analysis	Nucleus-Level Analysis	FOV-Grouped Analysis
Evaluation units	154 nuclei from 51 FOVs	51 source FOVs used as the primary evaluation units (26 Early, 25 Late)
Cross-validation protocol	Five-fold nucleus-stratified nested cross-validation; FOV-cluster bootstrap used for confidence intervals	Primary five-fold FOV-grouped nested cross-validation; zero FOV overlap between training and test sets
Classification accuracy	0.597 (95% CI: 0.510–0.689)	0.529 (95% CI: 0.392–0.667)
Precision	0.486 (95% CI: 0.392–0.600)	0.516 (95% CI: 0.400–0.630)
Recall	0.600 (95% CI: 0.477–0.745)	0.640 (95% CI: 0.440–0.800)
F1-score	0.537 (95% CI: 0.451–0.629)	0.571 (95% CI: 0.429–0.692)
ROC-AUC	0.623 (95% CI: 0.520–0.731)	0.586 (95% CI: 0.420–0.743)
Average precision	0.481 (95% CI: 0.391–0.638)	0.570 (95% CI: 0.463–0.762)
Brier score	0.242 (95% CI: 0.212–0.274)	0.256 (95% CI: 0.229–0.287)

**Table 14:** Pooled FOV-level performance of the additional full-time-course modelling experiments. All methods used the Days 1–3 versus Days 4–6 definition and the locked FOV-grouped outer folds.

Method	Accuracy	Precision	Recall	F1-Score	ROC-AUC	Average Precision	Brier Score
ViT-B/16 fine-tuning	0.529	0.516	0.640	0.571	0.586	0.570	0.256
LoRA ViT-B/16	0.510	0.500	0.520	0.510	0.531	0.526	0.260
Frozen ViT neural pooling	0.569	0.552	0.640	0.593	**0.614**	**0.586**	0.273
Hybrid morphology-gated attention MIL	**0.588**	**0.559**	**0.760**	**0.644**	0.597	0.567	0.296

**Table 15:** Pooled FOV-level performance of the DINOv2 multi-view ensemble for the extreme-timepoint sensitivity task.

Metric	Result
Source FOVs	30
Accuracy	0.767
Precision	0.714
Recall	0.769
F1-score	0.741
ROC-AUC	0.765
Average precision	0.753
Brier score	0.197
Accuracy 95% CI	0.600–0.900
ROC-AUC 95% CI	0.552–0.937

**Table 16:** Sensitivity of FOV-level performance to equal day representation. The primary analysis used six source FOVs from each of Days 1–6 (36 FOVs per resample), while the DINOv2 extreme-timepoint analysis used six FOVs from each of Days 1, 2, 5, and 6 (24 FOVs per resample). Balanced values are medians with 2.5th–97.5th percentile ranges across 20,000 resamples.

	Primary ViT-B/16		DINOv2 Extreme-Timepoint	
Metric	Original	Balanced	Original	Balanced
Accuracy	0.529	0.528 (0.444–0.611)	0.767	0.750 (0.708–0.833)
Precision	0.516	0.522 (0.455–0.591)	0.714	0.750 (0.692–0.833)
Recall	0.640	0.611 (0.556–0.722)	0.769	0.750 (0.750–0.833)
F1-score	0.571	0.564 (0.488–0.650)	0.741	0.750 (0.720–0.833)
ROC-AUC	0.586	0.583 (0.494–0.679)	0.765	0.750 (0.701–0.833)
Average precision	0.570	0.587 (0.526–0.719)	0.753	0.774 (0.720–0.867)
Brier score	0.256	0.256 (0.236–0.270)	0.197	0.213 (0.162–0.243)

## Data Availability

The microscopy nucleus dataset analysed in this study was acquired from cultured human induced pluripotent stem cell-derived cardiomyocytes (iPSC-CMs) at the University of East London Bioscience Laboratory. The dataset, source code, and associated documentation are maintained within the University of East London research repository and will be made available in accordance with the University’s data-sharing policies upon reasonable request or following publication.
